# From the Modeling of Experiments to the Ab Initio Prediction of Rate Constants: Statistical Rate Theory for a Quantitative Understanding of Gas‐Phase Ion Chemistry

**DOI:** 10.1002/cphc.70362

**Published:** 2026-04-20

**Authors:** Thomas Auth, Konrad Koszinowski

**Affiliations:** ^1^ Institut für Organische und Biomolekulare Chemie Universität Göttingen Göttingen Germany

**Keywords:** ab initio calculations, gas phase reactions, mass spectrometry, rate constants, transition states

## Abstract

Statistical rate theory has long been used for the analysis of gas‐phase ion reactions. Traditionally, it has mainly served as a framework for fitting experimental data, often obtained with highly specialized instrumentation, and extracting quantities of interest, such as reaction threshold energies. With the progress in quantum chemical calculations and their ability to provide accurate energy profiles along reaction coordinates, the reliable ab initio prediction of rate constants of gas‐phase ion reactions appears to be within reach. Such predictions would be quite valuable, because they enable the direct comparison between the results from theory and standard mass‐spectrometric experiments and, thus, aid in the interpretation of the latter. In this review, we seek to answer the question of the extent to which accurate ab initio predictions of gas‐phase ion reaction rate constants have become feasible and can be used for routine applications. After covering the basics of statistical rate theory and giving an overview of important programs for rate calculations, we demonstrate and discuss the current state of the field for four different examples: the dissociation of the *n*‐butylbenzene radical cation, the dissociation of benzylpyridinium ions, the unimolecular reactivity of anionic organometallic complexes, and the reactivity of organometallic ions toward proton donors.

## Graphical abstract

For directly comparing experimental results on gas‐phase ion reactions with the predictions from quantum chemical calculations, the latter must first be converted into rate constants. This review addresses the question of whether current methods of statistical rate theory can accomplish this task reliably.

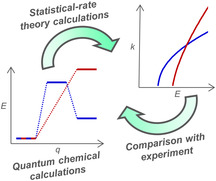

## 1 Introduction

Gas‐phase ion chemistry deals with uni‐ and bimolecular reactions of ions in the gaseous state. The importance of this subdiscipline of chemistry for understanding atmospheric [1, 2] and interstellar processes [3, 4] is obvious. However, its relevance goes far beyond these rather special fields and pervades the entire molecular sciences. One of the main reasons for the general pertinence of gas‐phase ion chemistry lies in the facility with which ions can be manipulated in the gaseous state [5, 6, 7, 8]. First, the application of electric and magnetic fields permits exquisite control of the motion of ions in the gas phase. Second, the low particle density implies that reactive collisions can be largely or virtually completely suppressed, thus enabling the preparation and probing of highly reactive species, which could not be handled in the condensed phase unless in low‐temperature matrices. Thanks to these features and the well‐defined experimental conditions achievable, gas‐phase ion techniques are very well‐suited for fundamental reactivity studies and the determination of thermochemical quantities. Since the 1960s, such studies have been systematically performed for numerous classes of ions [9, 10, 11, 12, 13, 14, 15, 16, 17, 18, 19, 20, 21, 22, 23]. More recently, tandem mass spectrometry has been applied to the structural analysis of proteins and other biomolecules and nowadays is used extensively and routinely for this purpose [24, 25, 26].

**FIGURE 1:**
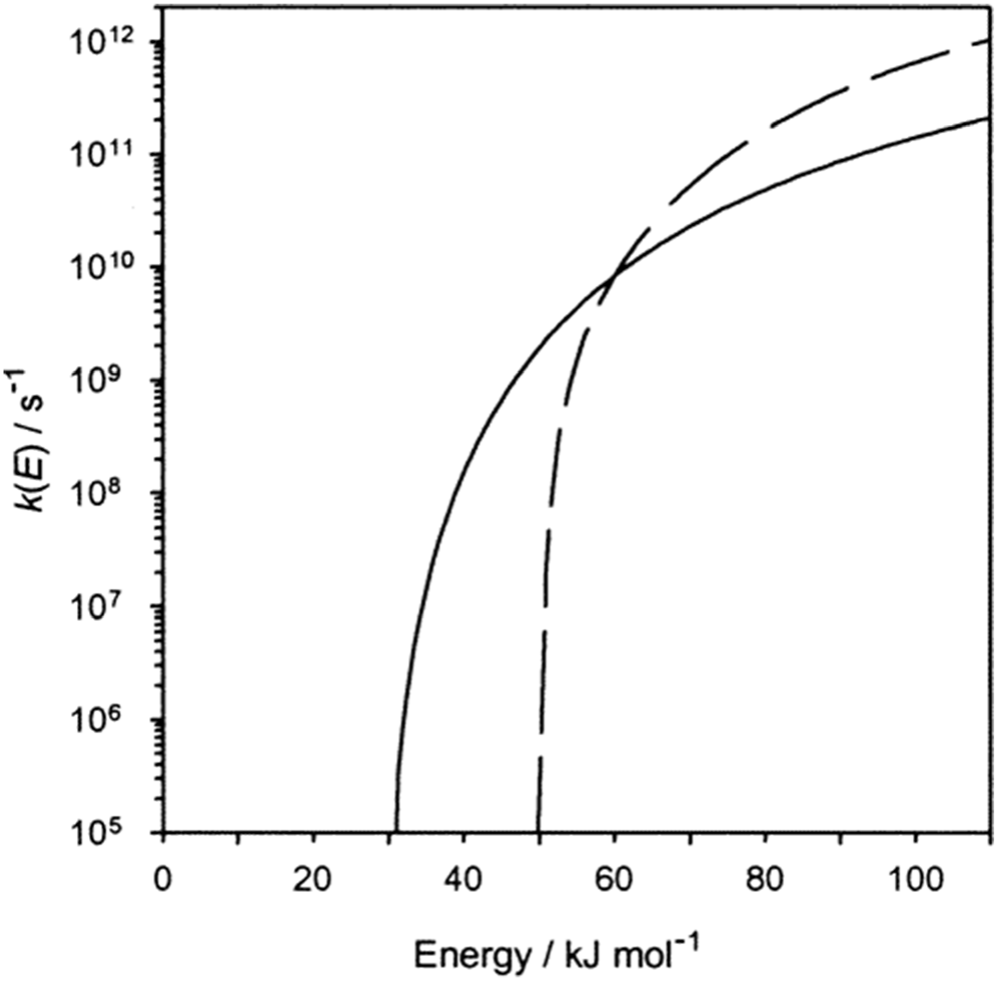
Schematic rate−energy curves showing the dependence of the unimolecular dissociation rate constant, *k*(*E*), on the available energy for two product channels, one with a tight TS with an activation entropy of Δ*S* ^‡^_1000_ = −0.4 J mol^−1^ K^−1^ and a threshold energy of *E*_0_ = 30 kJ mol^−1^ (solid line) and the other with a loose TS with Δ*S* ^‡^_1000_ = +18 J mol^−1^ K^−1^ and *E*_0_ = 50 kJ mol^−1^ (dashed line). Reproduced with permission from ref. [30]. Copyright 2001, American Chemical Society.

From the early gas‐phase ion experiments on, there has been a keen interest in a theoretical understanding of the underlying chemistry and physics. In the beginning, neither the experimental nor the theoretical methodology was sufficiently developed to attain such an understanding at a quantitative level. Nonetheless, statistical rate theory [27, 28] (for a definition, see Section 2) already then afforded valuable insight. A textbook example is given by the effect that the nature of the transition state (TS) [29] exerts on the rate constant of the corresponding reaction, *k*, and the energy dependence of the latter. For a so‐called tight TS, i.e., a structurally rigid TS, *k* is predicted to increase only relatively slowly as a function of the internal energy *E* of the reaction system. In contrast, *k*(*E*) rises much faster for a so‐called loose TS, i.e., a structurally less rigid TS (for a more specific definition of the two different types of TSs, see Section 2). As a consequence, the branching ratio between two competing reaction channels as a function of *E* can switch if they differ in the tightness of the involved TSs and their threshold energies (Figure 1) [30]. Understanding this behavior is of key importance for the correct interpretation of experiments probing such a system at different energies because, in this case, deviating branching ratios are to be expected and do not point to an error in one of the measurements.

**FIGURE 2:**
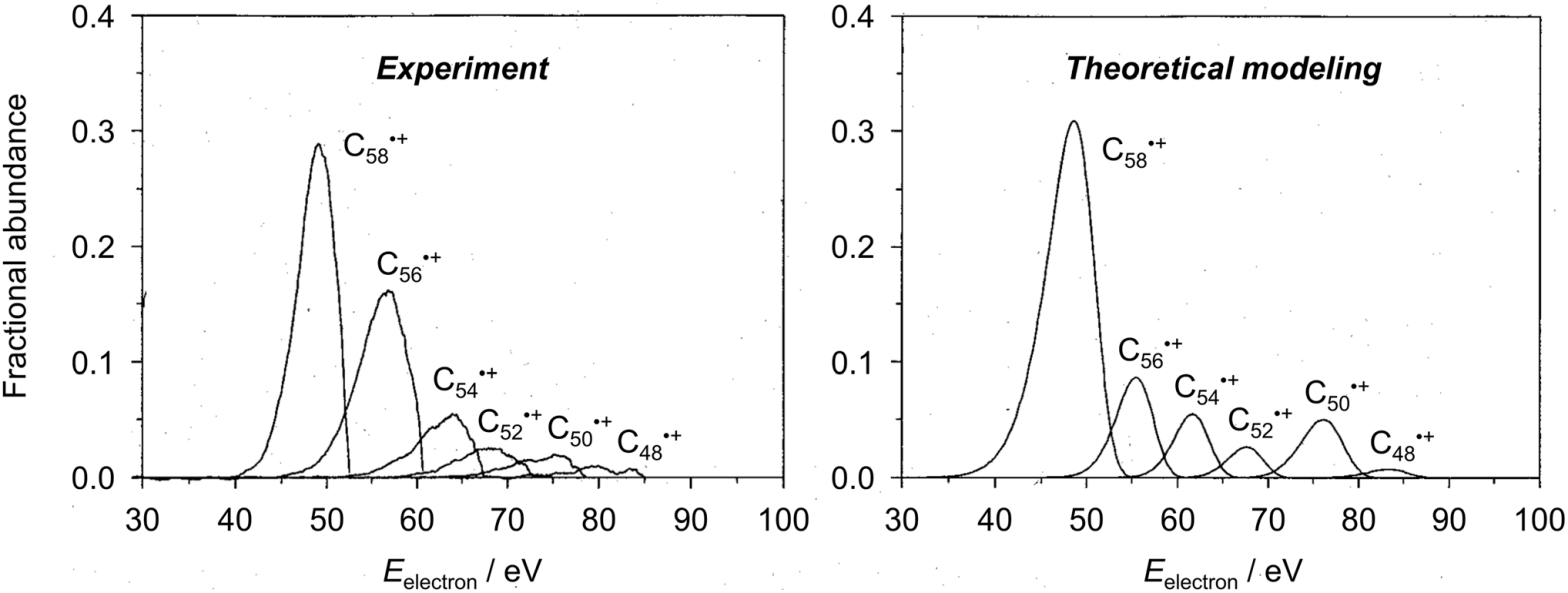
Breakdown curves for the fragmentation of fullerene C_60_ after electron ionization. Left: Experimental data. Right: Data modeled by means of statistical rate theory. Adapted with permission from ref. [36]. Copyright 1996, American Institute of Physics.

Later, advances both in experimental gas‐phase techniques and quantum chemical methods shifted the focus toward the goal of a quantitative understanding and modeling of the experimental data with the help of statistical rate theory [31]. The need for modeling, perhaps, is most important for measurements of reaction threshold energies, which form an important means for the determination of thermochemical quantities [32, 33, 34]. Fitting of the experimental data is required for finding the true onset of the monitored reaction, particularly if the signal‐to‐noise ratio is relatively poor. Furthermore, statistical‐rate theory calculations are essential for determining the so‐called kinetic shift, which corresponds to the difference between the observed appearance energy and the true threshold energy *E*_0_. Such a shift occurs when ions with internal energies above *E*_0_ do not react fast enough within the limited time window of the experiment to allow for the observation of the reaction products [35]. According to the predictions of statistical rate theory, the kinetic shift is largest for ions with many degrees of freedom, which render the accumulation of sufficient energy in the reactive mode less likely. Arguably, the most striking example of a kinetic shift is related to the fragmentation of fullerene C_60_ upon electron ionization. The appearance energies of C_58_^•+^ and further fragment ions resulting from the loss of C_2_ units after the initial ionization were found to be extremely high (> 43 eV) [36], which seemed to suggest an extraordinary stability of C_60_ (Figure 2, left). However, statistical‐rate theory modeling revealed that the high appearance energies mainly originated from very large kinetic shifts due to the large size of these cluster ions and the corresponding high number of degrees of freedom [36, 37]. The successful quantitative explanation of the experimental findings (Figure 2, right) is testament to the power of statistical rate theory and the underlying quantum chemical calculations. It also highlights the imperative need for considering the predictions of the former in the interpretation of the experimental results.

Quite often, the groups specializing in quantitative gas‐phase ion experiments not only built the necessary customized instrumentation themselves but also developed their own programs for performing statistical‐rate theory calculations for analyzing and modeling the obtained data (also see Section 3.1). Thus, both the preparation of the experiment and the data analysis required special expertise. This situation has changed more and more. Although experiments with careful energy control of the ions still need home‐built apparatus [38], commercial mass spectrometers have become extremely robust and today can be employed at least for simpler gas‐phase experiments without an in‐depth knowledge of all technical details [7, 23]. For the statistical‐rate theory calculations, the user likewise can choose from a variety of freely available software, whose operation does not require extensive experience in scientific computing (see Section 3). Finally, the progress in quantum chemical calculations has been immense [39, 40, 41, 42]. Such calculations are needed for predicting rotational constants and vibrational frequencies, which serve as input parameters for the statistical‐rate theory computations. A further input parameter for each considered elementary reaction is the threshold energy. For a long time, quantum chemical methods were not able to predict these energies with acceptable accuracy (Δ*E*  ≲ 10 kJ mol^−1^), but for rather small systems. The superior performance of modern quantum chemical approaches [43, 44] suggests that reaction energies and barriers for medium‐sized or even relatively large chemical systems can be computed with sufficient accuracy to use them for statistical‐rate theory predictions. Accordingly, today's users not only can rely on commercially available refined instrumentation but also have all theoretical tools for the ab initio calculation of rate constants at their command. As Glowacki and coworkers wrote already in 2012 [45]: “As … electronic structure theory is increasingly reliable, and as our fundamental understanding of energy transfer improves, we envision … routine and reliable prediction of nonequilibrium kinetics in arbitrary systems.”

In this review, we aim to answer the question of the extent to which this vision has already been realized for gas‐phase ion chemistry. We do so decidedly from the perspective of practitioners and seek to guide the experimentalist in the prediction of rate constants for comparison with the results of gas‐phase ion experiments, possibly performed with simple or only slightly modified commercial instruments. Such comparisons test the statistical‐rate theory calculations themselves as well as the underlying quantum chemical computations. The potential energy surfaces (PESs) obtained from the latter are of key importance for the understanding of chemical reactivity. As they elude a direct experimental determination, the only way to examine their accuracy experimentally lies in their conversion into reaction rate constants (or reaction cross sections) as experimentally accessible quantities. Thus, statistical‐rate theory calculations are indispensable for checking the validity of mechanistic hypotheses and benchmarking quantum chemical methods alike.

In the following, we will first address the basics of statistical rate theory and confine the discussion to qualitative explanations of the different approaches and their application to gas‐phase ion chemistry. Numerous previous reviews related to this topic discuss fundamental aspects of statistical rate theory in more detail [34, 46, 47, 48, 49, 50, 51, 52, 53, 54, 55, 56, 57]. We will then present several available programs for statistical‐rate theory calculations and focus on those that are suitable for treating gas‐phase ion reactions. In the main part of this review, we will showcase different applications of statistical‐rate theory calculations for the analysis of gas‐phase ion chemistry. Specifically, we will deal with the following reactions, which exhibit an increasing degree of complexity:

(i) Fragmentation of the *n*‐butylbenzene radical cation: A well‐studied model system, which serves as an example of the use of statistical‐rate theory calculations for the fitting of experimental data.
(ii) Fragmentation of benzylpyridinium ions: The dissociation of these so‐called thermometer ions has been investigated extensively because it provides information on the energies deposited into the ions in a given experiment.
(iii) Fragmentation of organometallic anions: These reactions correspond to examples for which rate constants have been predicted ab initio. The reactant ions are of considerable complexity and are of interest due to their practical importance in the condensed phase.
(iv) Protonation of organometallic ions: These reactions form the bimolecular counterpart to those to be discussed in the preceding section. Again, rate constants have been computed ab initio.

Finally, we will briefly evaluate the different approaches of statistical‐rate theory calculations for gas‐phase ion reactions before concluding the review.

## 2 Basics of Statistical Rate Theory for Ab Initio Predictions

Statistical rate theory is a generic term for the theoretical framework that enables the calculation of rate constants *k* for a chemical reaction based on (i) the PES along the reaction coordinate and (ii) the fundamental assumption that the internal energy of the reactant(s) of each involved elementary step is statistically distributed among the available internal degrees of freedom [34, 49, 51, 58]. In this way, microcanonical rate constants, *k*(*E*), as a function of the internal energy *E* of the reaction system, or canonical rate constants, *k*(*T*), as a function of the temperature *T* of the reaction system, can be obtained. A key component of statistical rate theory is the concept of a TS associated with each elementary reaction [29, 48, 59, 60]. The overall formalism of statistical rate theory is independent of whether ions or neutrals participate in a chemical reaction. However, the charge state of the reactants can affect the nature of the involved TS as detailed below. Furthermore, reaction systems in mass‐spectrometric experiments usually do not represent a canonical ensemble at a given temperature, and thus, the calculation of *k*(*E*) values is required for predicting their kinetics [47, 49, 50].

A unimolecular process featuring a single TS, i.e., the simplest chemical reaction, is the ideal starting point to elaborate on the basics of statistical rate theory. For such an elementary reaction, Rice–Ramsperger–Kassel–Marcus (RRKM) theory [27, 61, 62], which is also referred to as microcanonical transition state theory (TST) [48] and can be regarded as equivalent to quasiequilibrium theory [28], provides the fundamental equation for calculating *k*(*E*) values:

(1) $$k\left(E\right)=\frac{\sigma \;N^{‡}\left(E-E_{0}\right)}{h\;\rho \left(E\right)}$$

where *N* ^‡^(*E* − *E*_0_) is the sum of states of the TS, *E*_0_ is the zero‐point corrected energy difference between the TS and the reactant, i.e., the activation energy of the reaction, *ρ*(*E*) is the density of states of the reactant, *σ* is the reaction degeneracy factor, and *h* is Planck's constant. In general, *k* is a function of both *E* and the angular momentum *J*, meaning that Equation (1) is the simplified RRKM expression that holds for *J* = 0. As the reactant corresponds to a well‐defined configuration, viz., the energy minimum along the reaction coordinate, obtaining *ρ*(*E*) is straightforward: to this end, only the vibrational frequencies associated with the equilibrium structure of the reactant are needed [63, 64], which are accessible by means of quantum chemical calculations [40]. In contrast, there are different ways to determine *N* ^‡^(*E* − *E*_0_). Moreover, the possible options depend on the nature of the TS.

**FIGURE 3:**
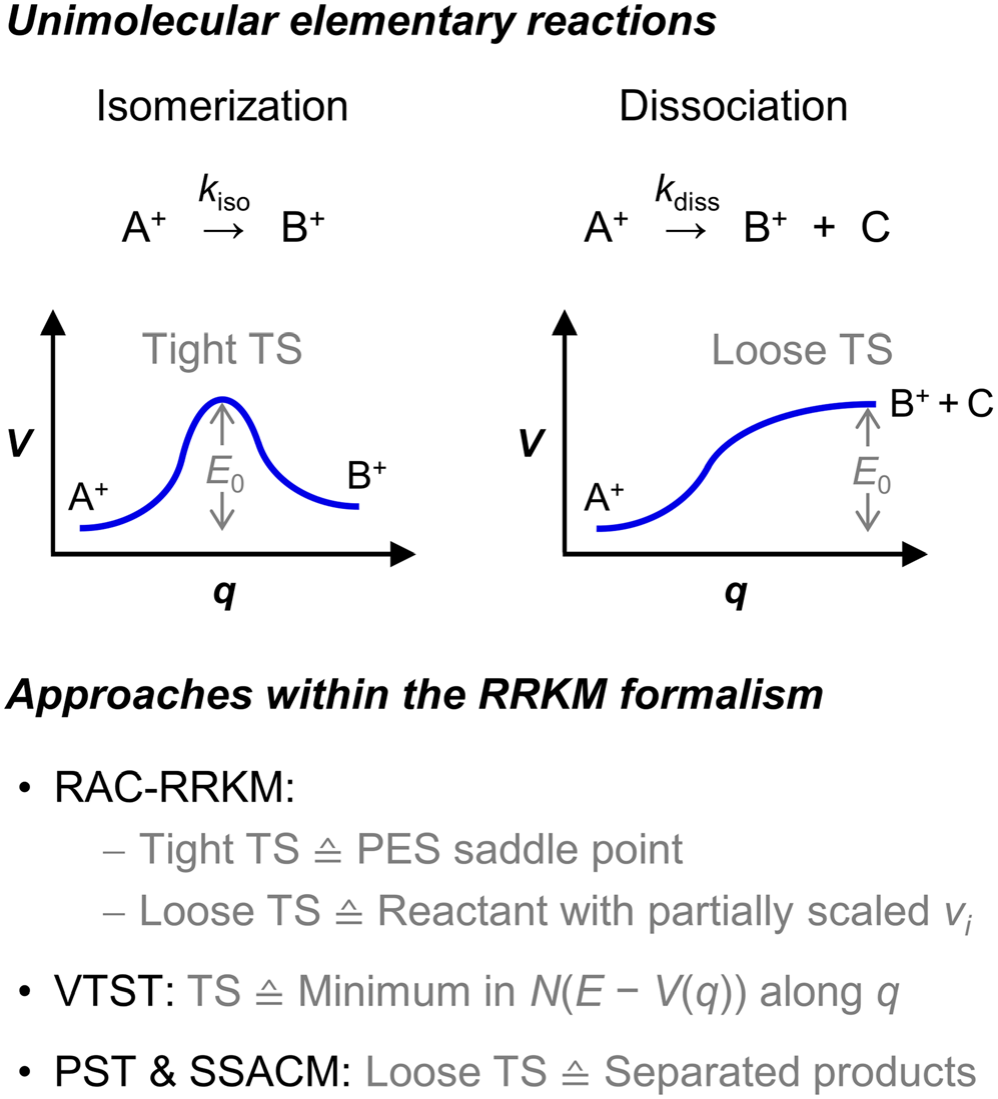
Representative potential energy (*V*) profiles for unimolecular elementary reactions (isomerization and dissociation) of molecular ions (top) and overview of different approaches for the calculation of microcanonical rate constants *k*(*E*) for these reactions within the RRKM formalism (bottom; *ν*_*i*_: vibrational frequencies). For the definitions of the acronyms, see text.

When considering an isomerization reaction, the PES along the energetically most favorable reaction coordinate (minimum‐energy pathway, denoted as *q* in the following) features a first‐order saddle point (Figure 3, top), and for this condition, the TS is classified as tight. In this case, the TS can be defined as a rigid activated complex (RAC) corresponding to the configuration of the saddle point (RAC‐RRKM approach; Figure 3, bottom) [51, 65, 66]. Under this assumption, the computation of *N* ^‡^(*E* − *E*_0_) merely requires the vibrational frequencies of the saddle point (except for its imaginary frequency) and the *E*_0_ value, which are obtainable from quantum chemical calculations as well. For a dissociation reaction of an ionic compound, the PES along *q* is typically qualitatively different from that of an isomerization, viz., it lacks a barrier in the reverse direction because of the pronounced attractive ion‐neutral interaction between the resulting product fragments [34, 67]. In such a case, where the potential energy steadily increases toward the products (Figure 3, top), the involved TS is generally viewed as loose, and the question arises of how to define it, or rather how to calculate *N* ^‡^(*E* − *E*_0_).

The RAC‐RRKM approach offers two possibilities in this respect. One is to determine *N* ^‡^(*E* − *E*_0_) using the set of vibrational frequencies of the reactant in combination with two modifications, namely, the vibrational mode that corresponds to *q* is omitted and the vibrational frequencies of the transitional modes, i.e., the vibrational modes of the reactant that are not conserved along *q*, are scaled by a factor less than 1 (cf. Figure 3, bottom) [68, 69]. Alternatively, a specific configuration along *q* between the reactant and the separated products derived from quantum chemical calculations can be chosen as TS, and its vibrational frequencies then serve as input for the *N* ^‡^(*E* − *E*_0_) computation [70, 71]. However, for the aforementioned scaling factor or the location of the TS along *q*, which both define the looseness of the TS model and significantly influence the final *k*(*E*) values, the optimal choice for a particular system is not known a priori. Therefore, the value of the RAC‐RRKM approach is fairly limited for the accurate ab initio prediction of rate constants for barrierless dissociation reactions.

An improved method for the calculation of the sum of states of the TS, which, inter alia, provides a solution to the shortcoming of the RAC‐RRKM approach regarding loose‐TS reactions, is the microcanonical variant of variational transition‐state theory (VTST) [55, 72, 73]. Here, the TS is defined for each considered *E* above *E*_0_ in such a way that it corresponds to the configuration along *q* that is associated with the lowest *N*(*E* – *V*(*q*)) value (Figure 3, bottom). Consequently, VTST affords a systematic and general procedure for identifying a plausible TS and calculating the reaction rate at a given internal energy for a unimolecular elementary reaction. However, to determine *N*(*E* – *V*(*q*)) as a function of *E* and *q*, detailed information on the PES along *q* is required. Furthermore, in the case of a barrierless dissociation, special care must be taken with respect to the description of the transitional modes, which makes VTST a challenging method to deal with [69, 74]. A generalized version of VTST, especially developed for loose‐TS processes, is variable‐reaction coordinate (VRC) TST, which incorporates a multidimensional PES region in the variational TS search instead of a single reaction coordinate [75].

A further and more convenient option for the treatment of loose‐TS dissociation reactions within the framework of RRKM theory assumes that the TS is located at the PES along *q* where the product fragments are well separated, viz., at an infinite distance between them for *J* = 0 or at the centrifugal barrier for *J* > 0. Under this premise, the transitional modes are treated as rotations of the products, and thus, *N* ^‡^(*E* − *E*_0_) is computed simply on the basis of the vibrational and rotational properties of the equilibrium structures of the formed fragments, which are available from quantum chemical calculations. The outlined approach involves a so‐called orbiting TS and is commonly denoted phase‐space theory (PST; Figure 3, bottom) [76, 77, 78]. Strictly speaking, it explicitly conserves angular momentum in the course of a reaction. For this reason, Armentrout and coworkers have termed their PST‐like method without the angular‐momentum constraint phase‐space limit (PSL) TS model [68]. The latter comprises the loosest possible TS of a barrierless dissociation and hence is the opposite of the reactant‐based RAC‐RRKM approach, which includes the tightest TS definition.

In the context of numerous guided‐ion beam mass‐spectrometric studies on the fragmentation of cationic metal‐ligand complexes, it was demonstrated that the PSL‐TS model is the appropriate choice for modeling the dissociation kinetics of M—L bonds that feature a neutral ligand L bound to a metal cation M^+^ by predominantly electrostatic, i.e., noncovalent interactions [34]. However, for dissociation reactions of ionic systems cleaving a covalent bond, PST‐like methods were shown to predict too steep *k*(*E*) curves. This finding indicates that the supposed orbiting TS overestimates the looseness of the true TS in those cases [69, 70]. One of the reasons for this overestimation is the fact that PST in general does not account for an inward shift of loose TS along *q* toward higher rigidity with increasing *E*. As is known from VTST investigations, this behavior is characteristic of most barrierless dissociations and results from an increasing anisotropy of the PES with decreasing product fragment separation [69, 79]. A conceptually straightforward remedy for the aforementioned shortcoming of PST is the simplified statistical adiabatic channel model (SSACM; Figure 3, bottom) [80]. Here, *N* ^‡^(*E* − *E*_0_) from PST is multiplied by an energy‐dependent rigidity factor *f*_rigid_(*E*), which yields *k*(*E*) curves in accordance with both the much more elaborate SACM approach [81] and VTST for a properly chosen *f*_rigid_(*E*). Unfortunately, no universal procedure (except for a full SACM treatment) for deriving appropriate *f*_rigid_(*E*) values for any loose‐TS dissociation has been established yet, but Troe and coworkers have provided some guidelines for estimating specific rigidity factors [52].

**FIGURE 4:**
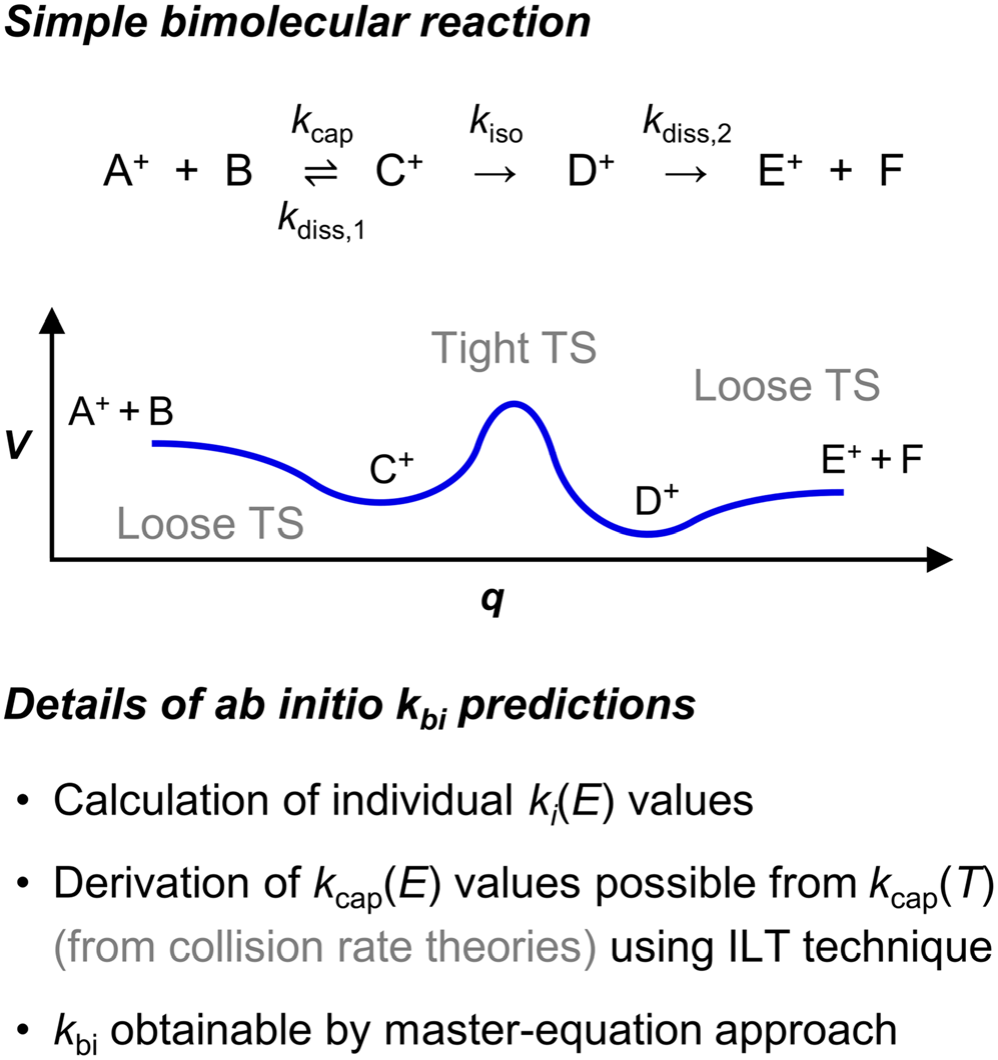
Representative potential energy (*V*) profile for a simple exothermic ion‐molecule reaction featuring reactant association (ion‐molecule capture), bond rearrangement (isomerization), and dissociation of the product ion‐molecule complex (top) as well as details of the typical methodology for calculating the overall bimolecular rate constant *k*_bi_ (bottom; ILT: inverse Laplace transform).

So far, we have detailed how ab initio *k*(*E*) values of a single unimolecular reaction step can be calculated by the RRKM formalism (Equation (1)). These calculations are already capable of modeling the kinetics of collision‐induced fragmentations of gaseous ions in mass‐spectrometric experiments (owing to the fact that these reactions are inherently irreversible) in the following two cases:

(i) Dissociations directly proceeding via a loose TS, such as direct bond cleavages (for examples, see Sections 4.1–4.3).
(ii) Two‐step reactions consisting of an isomerization, such as a bond rearrangement, and a subsequent dissociation, in which the energy barrier of the isomerization step is distinctly higher than the energy of the separated products (e.g., the energy profile for the sequence from C^+^ to E^+^ + F in Figure 4, top; also see Section 4.3). Here, the first step, as well as its reverse process, occurs considerably more slowly than the final one, meaning that each isomerization is immediately followed by the dissociation. Thus, the rate of the product formation is determined by *k*_iso_(*E*).

However, microcanonical rate constants from RRKM theory can only fully simulate the kinetics of these simple collision‐induced dissociation (CID) reactions as long as complicating effects due to interfering collisional activation/deactivation of collisionally activated ions can be excluded.

For multistep unimolecular fragmentation channels featuring one or more reversible isomerization steps before the final dissociation (e.g., a two‐step reaction, in which the threshold energy of the isomerization is lower than that of the subsequent dissociation), the prediction of their overall *k*_uni_(*E*) values at first requires computing *k*_*i*_(*E*) of each forward and backward step individually. Due to the lack of a *k*_*i*_(*E*)‐dependent analytical expression for *k*_uni_(*E*) in this case, the latter needs to be derived from the theoretical time evolution of the product state population for a given *E*, which can be obtained by solving the master equation of the reaction system, i.e., by numerical integration of the corresponding *k*_*i*_(*E*)‐dependent set of first‐order differential rate equations [82, 83]. This more demanding procedure is also necessary for the ab initio calculation of bimolecular rate constants (*k*_bi_) for gas‐phase ion‐molecule reactions [51]. Even in their simplest form, they comprise multiple steps, viz., the formation of the initial ion‐molecule complex, known as ion‐molecule capture, a bond rearrangement as the actual chemical reaction, and the final dissociation of the product ion‐molecule complex (Figure 4, top). Because of the significant ion‐neutral attraction between the reactants and products (see above), both ion‐molecule capture and dissociation are typically barrierless with respect to their PES, which leads to a so‐called double‐well potential with a single energy barrier for the overall bimolecular process. If the loose‐TS dissociation of the product ion‐molecule complex for such a simple bimolecular reaction is much faster than the tight‐TS isomerization step in the forward and backward direction (Figure 4, top; cf. the two‐step unimolecular reaction discussed above), the isomerization can be regarded as irreversible and the decomposition as occurring instantaneously. To determine *k*_bi_ with the help of the master‐equation approach in this instance, it is therefore only mandatory to take into account *k*_*i*_(*E*) of the ion‐molecule capture, the reverse dissociation, and the bond rearrangement toward the products.

While the calculation of the bimolecular rate constants *k*_cap_(*E*) of the loose‐TS ion‐molecule capture through the RRKM formalism is rather cumbersome [84], an alternative and more convenient strategy relies on canonical capture rate constants *k*_cap_(*T*) (Figure 4, bottom). For an ion‐molecule reaction in the gas phase, *k*_cap_(*T*) can be estimated easily by means of well‐established collision rate theories [85]. For example, the classical Langevin model [86] yields rate constants of ion‐molecule capture assuming that the interaction potential between the reactants corresponds to that between a point charge and an induced dipole (defined by the ion's charge and the molecule's polarizability *α*), which leads to the same *k*_cap_ for all *T*. If the collisions between ions and neutrals with permanent dipole moments *μ*_D_ are to be described, more refined approaches are required. Both the so‐called average dipole orientation (ADO) theory [87, 88] and the Su‐Chesnavich model [89] consider the interaction between the ion and the total dipole moment of the neutral, which results from a permanent contribution and that induced by the charge of the ion. For estimating this interaction, the relative orientation of the dipole moment is of key importance. While ADO theory assumes an average orientation for a given ion‐neutral distance (which depends on the ratio *μ*_D_/*α* ^1/2^ and *T*), the Su‐Chesnavich model makes use of classical trajectory calculations for simulating ion‐molecule capture collisions. From the results of these calculations, empirical expressions for *k*_cap_(*T*) have been derived. Proceeding from the obtained *k*_cap_(*T*), the inverse Laplace transform technique [90, 91] allows for the determination of the associated *k*_cap_(*E*) values (Figure 4, bottom) and, moreover, the microcanonical reaction rate constants of the backward dissociation, *k*_diss,1_(*E*), by taking into account the equilibrium constant of the ion‐molecule complex formation, which is available from quantum chemical calculations. Accordingly, it is generally possible to arrive at *k*(*E*) values for a barrierless dissociation step starting from the corresponding *k*(*T*) or that of the reverse association process.

Results for *k*_cap_(*E*), *k*_diss,1_(*E*), and *k*_iso_(*E*) are sufficient to determine overall microcanonical rate constants for the considered ion‐molecule reaction (cf. Figure 4, bottom) with the master‐equation approach, in which the assumption of pseudo‐first‐order reaction conditions is commonly used for bimolecular reactions [45, 83]. However, *k*_bi_(*E*) values themselves cannot be compared directly with rate constants of ion‐molecule reactions from mass‐spectrometric measurements. First, the internal energy of the reactants and consequently of the ion‐molecule intermediates corresponds to a distribution, which was found to be thermal‐like in quadrupole‐ion trap instruments [92, 93, 94]. This issue can be addressed by including a Boltzmann distribution of internal energies, *P*_*T*_(*E*), for the reactants and thereby a mixture of microcanonical ensembles within the master‐equation approach [45, 83]. Second, if a buffer gas is present (as is the case in quadrupole‐ion traps), collisional activation/deactivation of the formed ion‐molecule complexes can take place before they undergo backward dissociation or isomerization toward the products, for which reason the internal energy of the individual reaction intermediates and hence, the *k*_*i*_(*E*) values cannot be presumed to be conserved quantities. For estimating the evolution of the internal energy in time within the master‐equation formalism, ion‐buffer gas collision frequencies, *ν*_coll_, are predicted and fed into a phenomenological model for collisional energy transfer [45, 51, 95].

Although measured *k*_bi_ values for ion‐molecule reactions in quadrupole‐ion traps, as well as their calculated counterparts, refer to conditions where the reactants are thermalized because of collisions with the buffer gas and, thus, are temperature‐dependent, they are typically not equivalent to canonical *k*_bi_(*T*) values. This equivalence would hold only if all intermediates along *q* effectively featured a constant *P*_*T*_(*E*) during the reaction. Such a scenario could be reached at sufficiently high pressures of the buffer gas and collisional thermalization of all intermediates, i.e., when *ν*_coll_ is substantially larger than the *k*_*i*_(*E*) values of the unimolecular steps of the ion‐molecule reactions [96, 97, 98]. For a fair comparison between experimental and theoretical bimolecular rate constants, it is crucial to know the actual temperature and pressure of the buffer gas in order to incorporate these critical quantities in the master‐equation computation of *k*_bi_ values. In the case of conventional CID experiments, the reaction conditions are less well defined than for ion‐molecule reactions in quadrupole‐ion traps, viz., *P*(*E*) of mass‐selected reactants arising from collisional activation is unknown and even time‐dependent through the occurrence of multiple consecutive collisions [99, 100]. Furthermore, these experiments themselves do not furnish *k*_uni_ values but essentially only formation yields of products as a function of an acceleration voltage applied to the precursor ions, and therefore product branching ratios if multiple reaction channels exist. By determining those branching ratios dependent on *E* or *P*(*E*) (estimated or modeled) of the reaction system on the basis of calculated unimolecular *k*_*i*_(*E*) values, a meaningful comparison between the experimental outcome and the results from statistical rate theory is feasible.

As is evident from the above, accurately computing *k*(*E*) values for gas‐phase ion elementary reactions and predicting *k*_uni_ and *k*_bi_ values that can be compared to mass‐spectrometric measurements is a challenging task. In the following, we outline a few more aspects relevant for the precise calculation of microcanonical reaction rate constants using the RRKM formalism. For the sake of simplicity, our discussion has so far neglected the treatment of angular momentum in *k*(*E*) calculations by focusing on the special case of *J* = 0 (cf. Equation (1)). While approximating *k*(*E*) with *k*(*E*,*J *= 0) can be justified if angular momentum effects are insignificant, *k*(*E*) in fact corresponds to an average over *k*(*E*,*J*) values for the actual *J* distribution of the reactant at the considered internal energy, i.e., ⟨*k*(*E*,*J*)⟩_*J*_ for *P*_*E*_(*J*) [29, 101, 102]. To obtain the individual *k*(*E*,*J*) values via the RRKM equation, the rovibrational sum of states of the TS and rovibrational density of states of the reactant, *N* ^‡^(*E*,*J*) and *ρ*(*E*,*J*), respectively, have to be employed, which both directly account for the *J*‐dependent amount of *E* that is occupied due to rotation. A possible way to derive ⟨*k*(*E*,*J*)⟩_*J*_ from the associated *k*(*E*,*J*) set assumes a statistical *P*_*E*_(*J*), which includes an *E*‐dependent maximum *J* value and is defined by *ρ*(*E*,*J*) [34, 68].

Aside from discrepancies between supposed and real *P*_*E*_(*J*) of a reaction system, there are several further sources of error that can impair the quality of *k*(*E*) predictions. One is the description of the rovibrational degrees of freedom of the relevant molecular configurations as foundation for evaluating *N* ^‡^(*E*,*J*) and *ρ*(*E*,*J*), which is usually done within the rigid‐rotor harmonic oscillator approximation [58]. The latter, for instance, becomes rather problematic when normal modes resemble internal rotations or when anharmonic effects on vibrations are substantial, and hence, going beyond the harmonic‐oscillator model is required in these cases [51]. Other reasons for shortcomings in RRKM computations of *k*(*E*) values could be the neglect of tunneling corrections [103] or just the consequence of failure of the basic principles of statistical rate theory. The more frequently recrossing of the TS occurs, the more the RRKM approach overestimates the true *k*(*E*) (if other errors are insignificant) [48]. Additionally, the more the examined system deviates from ergodic behavior, meaning from a situation where the internal energy at a given *J* is randomly distributed over all accessible rovibrational states, the larger errors must be expected from RRKM theory [56]. Classical trajectory calculations, i.e., a specific type of molecular dynamics simulations, as an alternative tool for predicting chemical reactivity, can be used to reveal and quantify recrossing and nonstatistical characteristics of a reaction step [104].

Finally, it is important to point out that two major factors determine the accuracy of *k*_uni_ and *k*_bi_ values from statistical‐rate theory calculations, namely the suitability of the applied kinetic model and the validity of the employed data from quantum chemical calculations, i.e., geometries, vibrational frequencies, and electronic energies of the involved equilibrium and TS structures. Accordingly, quantitative differences between experimental and theoretical rate constants can have various origins, so that their comparison does not strictly allow the error assessment of the individual contributions of the ab initio value.

## 3 Software for Statistical‐Rate Theory Calculations

In this section, we give an overview of software that is suitable for statistical‐rate theory calculations on gas‐phase ion reactions. As such, these programs can perform microcanonical calculations and specifically model barrierless elementary reactions. We focus on software that is intended for dissemination and whose provision is an invaluable service to the gas‐phase ion chemistry community. To facilitate the discussion, we ordered the presented software into three different groups. The first two groups comprise programs whose use for statistical‐rate theory calculations on gas‐phase ion reactions is well‐established and which are primarily designed for the modeling of experimental data or the prediction of rate constants on the basis of results from quantum chemical calculations. For each of these programs, we give a short description of its main applications as well as information on its release and its main capabilities in terms of statistical‐rate theory calculations for gas‐phase ion chemistry. The third group includes statistical‐rate theory software, which has not yet been frequently used or is not ideally suited for gas‐phase ion reactions due to not meeting the abovementioned criteria, and in addition, software for predicting gas‐phase ion reactions, which relies on a molecular dynamics approach.

### 3.1 Software for Modeling Gas‐Phase Ion Chemistry Experiments

#### 3.1.1 CRUNCH

• **Description**: Program by Armentrout and coworkers for analyzing and modeling energy‐resolved reaction cross sections to derive threshold energies with the help of statistical‐rate theory methods.
• **Release**: Armentrout and coworkers first mentioned CRUNCH in a publication from 1997 [68] (approx. 450 citations [105]). This work, together with three follow‐up articles [34, 106, 107], describes the features of the program, including the implemented statistical‐rate theory models. The program has been continuously updated.
• **Main capabilities for statistical‐rate theory calculations for gas‐phase ion chemistry**: Calculation of *k*(*E*) values (including the consideration of angular momentum) for elementary reactions using the RAC‐ or PSL‐RRKM approach.
• **Additional information**: On the basis of CRUNCH, Chen and coworkers developed the program **L‐CID** [108] (Ligand Collision‐Induced Dissociation, presented in 2007, approx. 80 citations [105]) and later provided the auxiliary Python code **unikin** [71] (2021, approx. 10 citations [105], publicly available via the DOI of the article), which calculates *k*(*E*) values (including the consideration of angular momentum) for unimolecular elementary reactions by means of the RAC‐RRKM, PSL‐RRKM, VTST, or SSACM approach.

#### 3.1.2 MassKinetics

• **Description**: Program by Vékey and coworkers for modeling and analyzing CID experiments (in‐source and MS^2^) with the help of statistical‐rate theory methods.
• **Release**: Drahos and Vékey introduced MassKinetics in a publication from 2001 [109] (approx. 150 citations [105]). Since the public release of its first version in 2001, the program has been continuously updated. The program is publicly available (https://masskinetics.software.informer.com, accessed January 2026).
• **Main capabilities for statistical‐rate theory calculations for gas‐phase ion chemistry**: Calculation of *k*(*E*) values (without the consideration of angular momentum) for unimolecular elementary reactions using the RAC‐RRKM approach (including a specific loose‐TS option for treating barrierless dissociations).

#### 3.1.3 PEPICO Modeling Software

• **Description**: Program by Baer and coworkers for modeling and analyzing PEPICO experiments with the help of statistical‐rate theory methods.
• **Release**: The program had been in use for more than ten years before Sztáray, Bodi, and Baer first described it in 2010 [110] (approx. 150 citations [105]). It has been further updated since then.
• **Main capabilities for statistical‐rate theory calculations for gas‐phase ion chemistry**: Calculation of *k*(*E*) values (including the consideration of angular momentum) for unimolecular elementary reactions by means of the RAC‐RRKM, PSL‐RRKM [111], VTST, or SSACM approach.

### 3.2 Software for the Prediction of Rate Constants

#### 3.2.1 Polyrate

• **Description**: Program by Truhlar and coworkers for the calculation of rate constants of elementary reactions by means of TST methods.
• **Release**: Since the release of its first version in 1987 [112], Polyrate has been continuously updated [113, 114, 115] (approx. 570 citations of the article presenting the Polyrate 4 version from 1992 [105]) and is publicly available (https://doi.org/10.5281/zenodo.8213312).
• **Main capabilities for statistical‐rate theory calculations for gas‐phase ion chemistry**: Calculation of *k*(*E*) values (including the consideration of angular momentum) for uni‐ and bimolecular elementary reactions by means of VTST and VRC‐TST approaches.
• **Additional information**: Polyrate 2023 can be interfaced with the program **TUMME 2023** [116] (Tsinghua University Minnesota Master Equation, 4 citations [105], publicly available via https://doi.org/10.5281/zenodo.7943283, also see below) to use the latter as a master‐equation solver for the calculation of *T*‐ and *p*‐dependent *k*_uni_ and *k*_bi_ values for single‐ and multistep reactions based on *k*(*E*) values from Polyrate (*T* and *p* refer to buffer gas).

#### 3.2.2 MultiWell

• **Description**: Program by Barker and coworkers for the calculation of rate constants of elementary and multistep reactions using statistical rate theory methods in combination with a master‐equation approach.
• **Release**: After the release of its first version in 1999, MultiWell was presented in a publication in 2001 [95] (approx. 560 citations [105]). The program has been continuously updated and is publicly available (https://multiwell.engin.umich.edu/downloads/, accessed January 2026).
• **Main capabilities for statistical‐rate theory calculations for gas‐phase ion chemistry**: Calculation of *k*(*E*) values (including the consideration of angular momentum) for uni‐ and bimolecular elementary reactions by means of RAC‐RRKM and inverse Laplace transform approaches, as well as of *T*‐ and *p*‐dependent *k*_uni_ and *k*_bi_ values for single‐ and multistep reactions based on *k*(*E*) values using a master‐equation approach (*T* and *p* refer to buffer gas).

#### 3.2.3 MESMER (Master Equation Solver for Multi‐Energy Well Reactions)

• **Description**: Program by Glowacki et al. for the calculation of rate constants of elementary and multistep reactions using statistical‐rate theory methods in combination with a master‐equation approach.
• **Release**: After the release of its first version in 2009, MESMER was described in an article in 2012 [45] (approx. 560 citations [105]). The program has been continuously updated and is publicly available (https://sourceforge.net/projects/mesmer/files/mesmer/, accessed January 2026).
• **Main capabilities for statistical‐rate theory calculations for gas‐phase ion chemistry**: Calculation of *k*(*E*) values (including the consideration of angular momentum) for uni‐ and bimolecular elementary reactions by means of RAC‐RRKM and inverse Laplace transform approaches, as well as of *T*‐ and *p*‐dependent *k*_uni_ and *k*_bi_ values for single‐ and multistep reactions based on *k*(*E*) values using a master‐equation approach (*T* and *p* refer to buffer gas).

#### 3.2.4 MESS (Master Equation System Solver)

• **Description**: Program by Georgievskii et al. for the calculation of rate constants of elementary and multistep reactions by means of statistical‐rate theory methods in combination with a master‐equation approach.
• **Release**: MESS was first released in 2016 and has been updated since then. The program is publicly available (https://tcg.cse.anl.gov/papr/codes/mess.html, accessed January 2026). While there is no article directly introducing the program, its key theoretical foundations were described in 2013 [117] (approx. 630 citations [105]).
• **Main capabilities for statistical‐rate theory calculations for gas‐phase ion chemistry**: Calculation of *k*(*E*) values (including the consideration of angular momentum) for uni‐ and bimolecular elementary reactions by means of RAC‐RRKM, PST, and VTST approaches, as well as of *T*‐ and *p*‐dependent *k*_uni_ and *k*_bi_ values for single‐ and multistep reactions based on *k*(*E*) values using a master‐equation approach (*T* and *p* refer to buffer gas).
• **Additional information**: Klippenstein and collaborators also developed two further programs complementary to MESS, namely **VaReCoF** (Variable Reaction Coordinate Flux) and **EStokTP** (Electronic Structure to *k* ( *T* , *P* )). The former, which should be cited together with an associated publication from 2005 [118] (approx. 200 citations [105]), can be used for performing VRC‐TST calculations for elementary reactions that involve a loose TS. The latter, presented in a publication in 2019 [119] (approx. 120 citations [105]), directly couples quantum chemistry software packages (Gaussian or Molpro) and VaReCoF with MESS. Both programs are publicly available (https://tcg.cse.anl.gov/papr/codes/varecof.html and https://github.com/EStokTP/EStokTP, accessed January 2026). Together, MESS and VaReCoF have superseded VariFlex [120], which offered comparable functionalities but is no longer publicly available.

### 3.3 Further Software

**TheRate** (Theoretical Rates): Program by Truong and coworkers for the calculation of canonical rate constants for barrier‐involving elementary reactions by means of TST/VTST approaches [121].

**KiSThelP** (Kinetic and Statistical Thermodynamical Package): Program by Henon and coworkers, which inter alia enables the calculation of canonical and microcanonical rate constants for barrier‐involving elementary reactions by means of TST/VTST approaches and RAC‐RRKM theory, respectively [122].

**Pilgrim**: Program by Ferro‐Costas et al. for the calculation of canonical rate constants for barrier‐involving elementary reactions by means of TST/VTST approaches and the simulation of multistep reactions based on the obtained *k*(*T*) values using the kinetic Monte Carlo method [123].

**TUMME** (also see above): Program by Zhang et al. for the calculation of *k*(*E*) values for elementary reactions and phenomenological rate constants for single‐ and multistep reactions by means of RAC‐RRKM, VTST, inverse Laplace transform, and master‐equation approaches [124].

**Arkane** (Automated Reaction Kinetics and Network Exploration:) Program by Grinberg Dana et al., which, inter alia, enables the calculation of *k*(*E*) values for elementary reactions and phenomenological rate constants for single‐ and multistep reactions by means of RAC‐RRKM, inverse Laplace transformations, and master‐equation approaches [125].

**VENUS**: This pioneering molecular dynamics program by Hase and coworkers can be employed to carry out classical trajectory calculations for reactive systems for various initial conditions [126]. Originally, analytical PESs were used for these computations, whereas by now, direct dynamics simulations are feasible through interfaces with quantum chemistry software [104].

**QCxMS** (Quantum‐Chemical x Mass Spectra): This molecular dynamics program by Grimme and coworkers is designed for the prediction of CID and electron‐ionization mass spectra. It allows for the execution of classical trajectory calculations on PESs computed "on the fly" with a semiempirical quantum chemical method and has the option to model collision processes between the precursor ion and the neutral collision gas explicitly [127].

**CIDMD** (Collision‐Induced Dissociation via Molecular Dynamics): This framework by Lee et al., like QCxMS, was developed for predicting CID spectra and relies on ab initio molecular dynamics simulations [128].

## 4 Examples of Statistical‐Rate Theory Calculations Applied to Specific Gas‐Phase Ion Reactions

### 4.1 Dissociation of the *n*‐Butylbenzene Radical Cation

**FIGURE 5:**
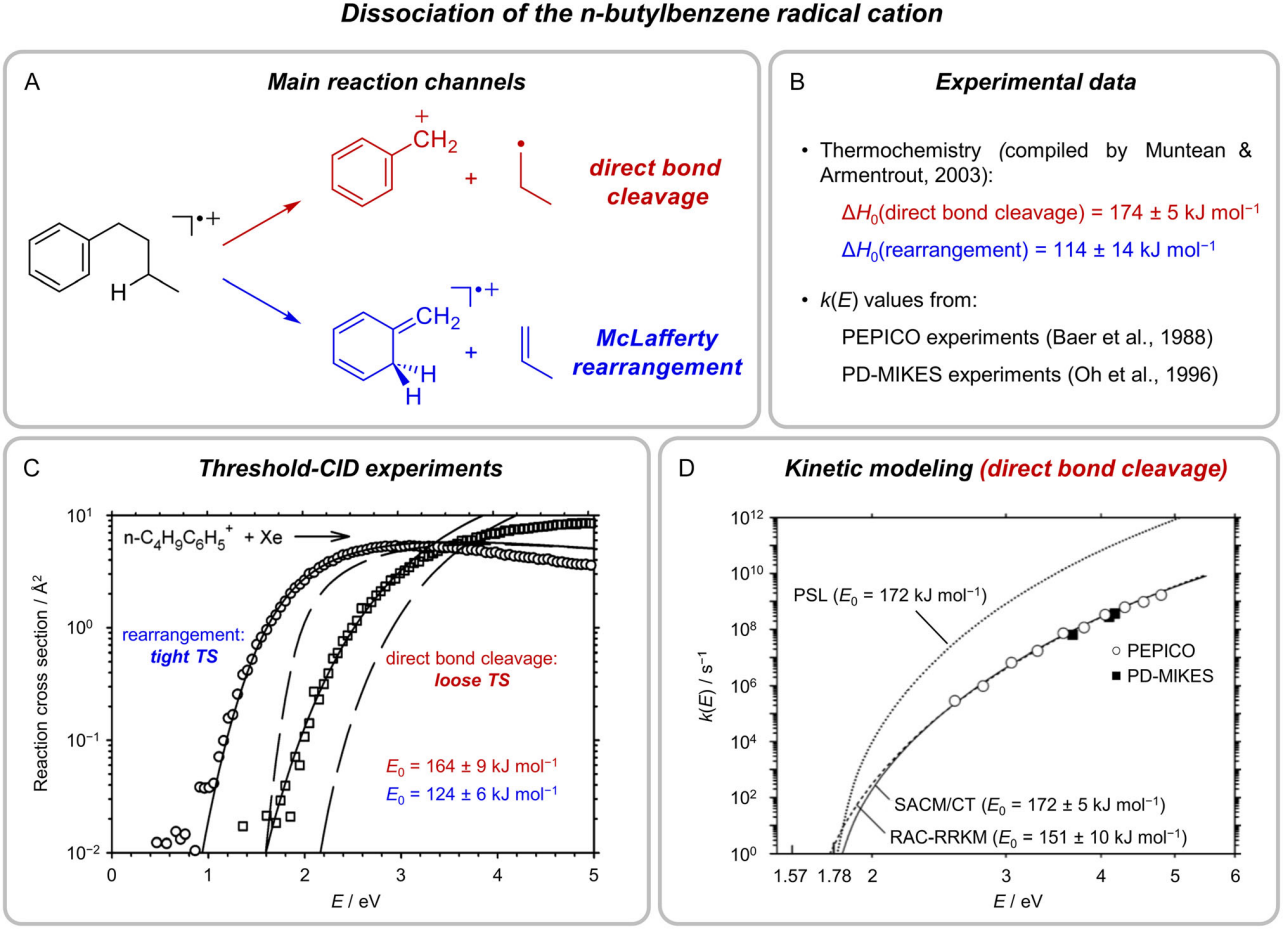
Low‐energy gas‐phase dissociation of the *n*‐butylbenzene radical cation. (A) Reaction channels. (B) Available key experimental data. (C) Energy‐resolved reaction cross sections determined by guided‐ion beam threshold collision‐induced dissociation experiments (extrapolated to zero Xe pressure, symbols) together with reaction cross sections modeled with the help of RAC‐RRKM calculations for reactants at *T *= 0 (dashed lines) and convoluted over their experimental kinetic and internal energy distributions (solid lines) [70]. (D) RAC‐RRKM (dashed line) and statistical adiabatic channel model/classical trajectory (SACM/CT) fits of the *k*(*E*) values for the direct bond cleavage obtained from PEPICO and PD‐MIKES experiments [80]. For comparison, a *k*(*E*) curve predicted by PSL‐RRKM [111] calculations is shown. Adapted with permission from refs. [70] and [80]. Copyright 2003 and 2007, American Chemical Society.

The unimolecular decomposition of the *n*‐butylbenzene radical cation is one of the best‐studied model systems in gas‐phase ion chemistry [70, 80, 129, 130, 131, 132, 133, 134, 135, 136]. The interest in this fragmentation arises mainly from the fact that it comprises two well‐defined, but very different low‐energy reaction channels: (i) the direct cleavage of the benzylic C—C bond to afford the benzyl cation and a propyl radical, as well as (ii) the elimination of propene resulting from a McLafferty rearrangement (Figure 5A). Given that the latter channel involves a tight TS and is energetically less demanding than the former proceeding via a loose TS, the branching ratio between the two pathways strongly changes as a function of energy. For both reaction channels, the reaction enthalpies Δ*H*_0_ can be derived from available standard heats of formation of the participating species (Figure 5B) [70]. Seminal photoelectron‐photoion coincidence (PEPICO) measurements by Baer et al. determined the branching ratio of the two competing reaction channels for ion internal energies of 2.0 ≤ *E *≤ 6.5 eV as well as rate constants *k*(*E*) of the rearrangement reaction for 1.5 ≤ *E *≤ 3.0 eV (Figure 5B) [134]. By modeling these rate constants with the RAC‐RRKM approach (see Section 2) using the threshold energy *E*_0_ and the TS vibrational frequencies as fitting parameters, *k*(*E*) values were extrapolated up to internal energies beyond 7 eV. Together with the measured branching ratios, these extrapolated values allowed for the derivation of rate constants of the direct bond cleavage. By a combination of photodissociation mass‐analyzed ion kinetic energy spectrometry (PD‐MIKES) experiments and RAC‐RRKM fits, Oh et al. obtained further *k*(*E*) values for both fragmentation pathways for 3.5 ≤ *E *≤ 4.5 eV, which agreed very well with the results from Baer et al. [135]

Later, Muntean and Armentrout probed the reaction system by energy‐resolved guided‐ion beam threshold‐CID measurements (Figure 5C) [70]. The recorded data directly shows the divergent energy dependence of the two reaction channels and, thus, provides a prototypical example of the differing behavior of reactions proceeding via tight and loose TSs (cf. Figure 1). Muntean and Armentrout also performed quantum chemical calculations to find the minimum‐energy paths of the reactions on the PES. By means of a relaxed surface scan, they proved the direct bond cleavage to be barrierless. Based on this feature, they first considered the PSL‐RRKM approach (see Section 2) for this channel and tested it for the *k*(*E*) data determined by Baer et al. [134] and Oh et al. [135] To this end, they employed the CRUNCH software (see Section 3.1) and used the geometries and vibrational frequencies from their quantum chemical calculations as input data. The resulting fit was of only poor quality and associated with a threshold energy of *E*_0_ = 222 kJ mol^−1^, which was significantly larger than the corresponding experimental Δ*H*_0_ reference value derived from the literature (174 ± 5 kJ mol^−1^, Figure 5B). This finding clearly showed that the PSL‐RRKM approach predicted a too small kinetic shift due to assuming too loose a TS in this case, even though the direct bond cleavage is a barrierless process. As an alternative, Muntean and Armentrout modeled the *k*(*E*) data with the RAC‐RRKM method. For the TS, they tested different configurations along the reaction coordinate defined by their relaxed surface scan and obtained the best result for a distance of *d *= 3.0 Å between the two carbon atoms of the dissociating bond. The corresponding fit reproduced the experimental rate constants well and yielded a threshold energy of *E*_0_ = 151 kJ mol^−1^. This value agreed more closely with the experimental reference (see above) than the result from the PSL‐RRKM calculations, but was somewhat too low, indicating that the RAC‐RRKM approach overestimated the tightness of the TS and predicted too large a kinetic shift. For the McLafferty rearrangement competing with the direct bond cleavage, Muntean and Armentrout neglected the multistep nature of the reaction and applied an RAC‐RRKM model, which incorporated merely the presumably rate‐determining C—C bond cleavage step. With this strategy, they were able to fit the experimental *k*(*E*) data from Baer et al. [134] and Oh et al. [135] fairly well and derived a threshold energy of *E*_0_ = 111 kJ mol^−1^ for the rearrangement channel in very good accordance with the experimental Δ*H*_0_ value for this reaction (114 ± 14 kJ mol^−1^, Figure 5B). However, for accomplishing this agreement, the authors had to increase the tightness of the considered TS by using an unusually high scaling factor for the vibrational frequencies, which most likely reflects the oversimplistic nature of the underlying kinetic model. With the aid of the optimized RAC‐RRKM approaches for the two competing dissociation reactions, Muntean and Armentrout were eventually able to fit the measured CID cross sections in the threshold region properly and thereby extracted quite accurate *E*_0_ values (Figure 5C), which emphasizes the robustness of their methodology as a whole.

Subsequently, Troe et al. revisited the dissociation of the *n*‐butylbenzene radical cation and analyzed the direct bond cleavage by means of the SACM approach combined with classical trajectory calculations [80]. For this purpose, they constructed an analytical anisotropic potential of reduced dimensionality along the reaction coordinate as basis for their transitional mode dynamics treatment. The radial part of the potential consisted of a short‐range Morse‐type component as well as a long‐range modified ion‐induced dipole component and could be fitted to the minimum‐energy path previously identified by Muntean and Armentrout [70]. More importantly, the anisotropy of the potential induces kinetic constraints, which find their expression in rigidity factors *f*_rigid_(*E*,*J*) in the SACM framework (cf. Section 2). Thus, these factors quantitatively describe the anisotropy‐induced increased tightness of the actual TS in comparison to that of the PSL model. Nevertheless, the TS of both the SACM approach and the PSL model would be qualitatively classified as loose. Considering an average quantum number of the total angular momentum of *J* = 87, Troe et al. could reproduce the *k*(*E*) values from Baer et al. [134] and Oh et al. [135] very well with the results from their SACM/classical trajectory calculations by adjusting the threshold energy *E*_0_ and an anisotropy parameter (Figure 5D). In excellent agreement with the Δ*H*_0_ value for the direct bond cleavage provided by Muntean and Armentrout [70] (see above), they obtained *E*_0_ = 172 ± 5 kJ mol^−1^, thus demonstrating the superior performance of the SACM approach. Remarkably, Troe et al. achieved a virtually identical fit with the SSACM method (see Section 2), which simply relied on an analytical expression for *f*_rigid_(*E*,*J*) as function of two parameters and rate constants from PSL‐RRKM calculations (for the latter, see Figure 5D). Specifically, this fit comprised rigidity factors in the range of 1 ≥ *f*_rigid_(*E*,*J*) ≥ 3.7·10^−3^ (for *E* being increased from *E*_0_ to high values).

Halbert and Bouchoux also took up the SSCAM approach for the direct bond cleavage [136]. With an energy‐independent rigidity factor of *f*_rigid_ = 10^−2^ and a theoretically predicted threshold energy of *E*_0_ = 180 kJ mol^−1^, they were able to model the experimental *k*(*E*) values from Baer et al. [134] and Oh et al. [135] quite well. Moreover, their quantum chemical calculations pointed to an additional relevant dissociation channel of the *n*‐butylbenzene radical cation, resulting in the formation of the tropylium ion together with the propyl radical. The threshold energy for this channel was computed to lie approx. 30 kJ mol^−1^ below that of the direct bond cleavage. However, as this reaction involves a complex mechanism including three rearrangement steps, it is kinetically disfavored compared to the competing McLafferty rearrangement and the direct bond cleavage, and contributes at best only very little to the observed fragmentation.

### 4.2 Dissociation of Benzylpyridinium Thermometer Ions

**FIGURE 6:**
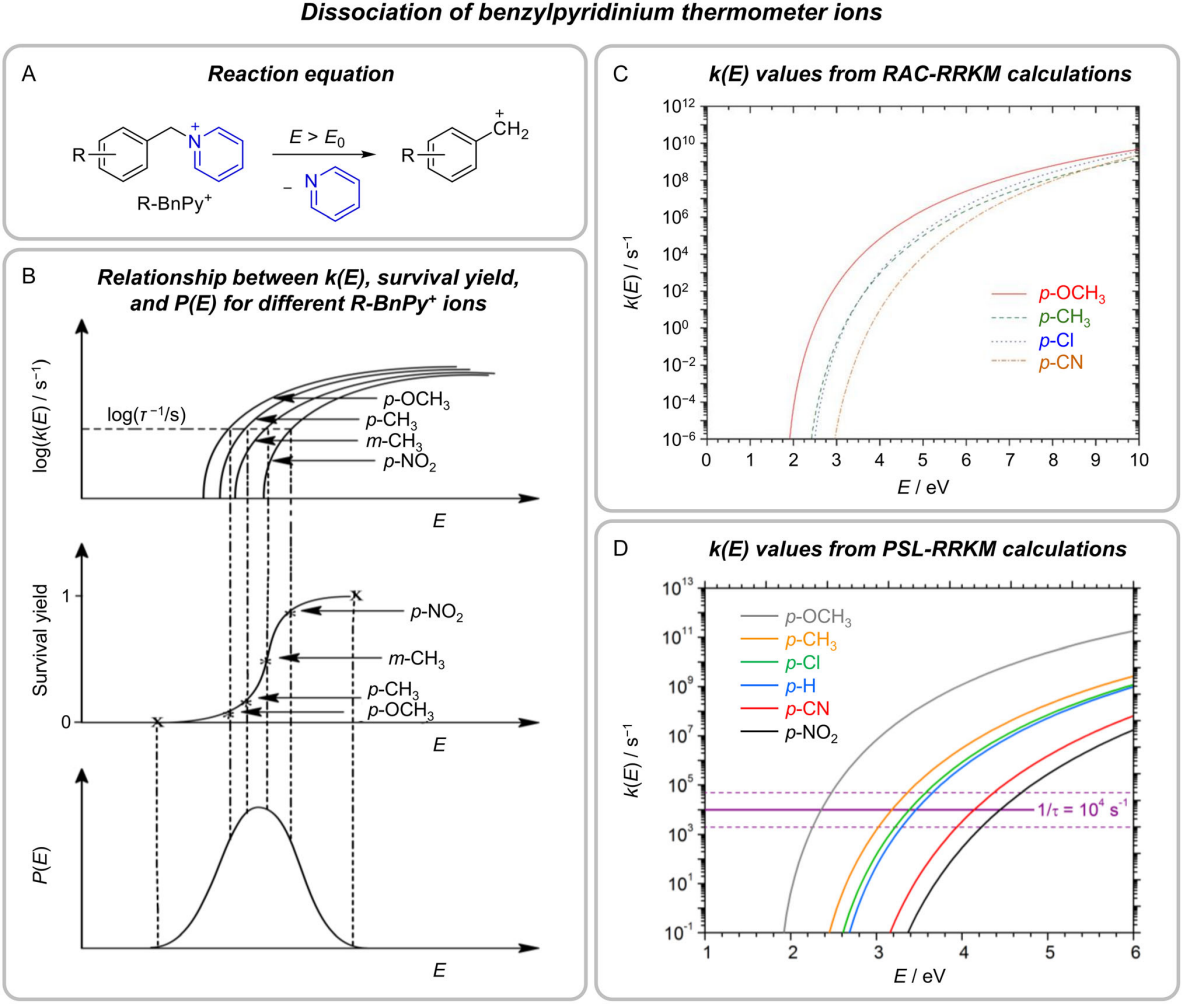
Gas‐phase dissociation of benzylpyridinium thermometer ions R‐BnPy^+^. (A) Reaction equation. (B) Relationship between dissociation rate constants *k*(*E*), survival yields (plotted against threshold energies *E*_0_ or appearance energies *E*_app_ of the dissociation of R‐BnPy^+^ ions), and internal‐energy distribution *P*(*E*) of the ions [139]. (C) Rate constants *k*(*E*) predicted by RAC‐RRKM calculations [140]. (D) Rate constants *k*(*E*) predicted by PSL‐RRKM calculations [141]. Adapted with permission from refs. [139, 140], and [141]. Copyright 2011, John Wiley & Sons, 2013 American Institute of Physics, and 2019, American Chemical Society.

In mass spectrometry and gas‐phase ion chemistry, the internal energy *E* of the investigated ions is of critical importance, as the discussion above has shown. For this reason, there is a keen interest in the experimental determination of this quantity. One of the most straightforward and popular ways of determining ion energies relies on so‐called thermometer ions. These ions dissociate in a well‐defined manner once their internal energies exceed the corresponding threshold energies *E*_0_ and thereby afford information on the probed experimental conditions [137, 138]. Typical examples are benzylpyridinium ions, which dissociate by the loss of pyridine (Figure 6A). As Morsa et al. have shown by IR‐multiphoton spectroscopy, the dissociation yields the benzyl cation as primary fragment ion [142]. Therefore, complicating effects due to a possible tropylium isomerization can be excluded. The introduction of substituents into the benzyl scaffold allows for the modulation of the stabilities of the resulting benzyl cations and, thus, of the *E*_0_ values of the Bn—Py bond dissociation. In the case of the *para*‐NO_2_‐ and ‐I‐substituted systems, competing fragmentation reactions compromise their usefulness as thermometer ions [143, 144].

Typically, the dissociation of a whole set of related thermometer ions, such as differently substituted benzylpyridinium ions, is considered, for which so‐called survival yields (fractions of undissociated thermometer ions) are determined [138, 139]. These survival yields are then plotted against the corresponding *E*_0_ values (Figure 6B, middle). The resulting curves show a sigmoidal shape and, upon derivation, afford the internal‐energy distribution of the ions *P*(*E*) (Figure 6B, bottom). Importantly, the outlined simple approach is only valid if the time scale of the experiment *τ* is sufficiently long such that all ions with *E *> *E*_0_ will dissociate within this period and no kinetic shift (see above) has to be accounted for. In most cases, the experimental time scale will not be long enough to exclude kinetic shifts. Thus, the typical procedure correlates the survival yields not with the threshold energies *E*_0_ but with the appearance energies *E*_app_. The latter are defined as the energies for which the dissociation reactions occur with a rate constant of *k*(*E*_app_) = *τ* ^−1^ and hence, become clearly observable (63% fragmentation yield) within the experimental time scale (Figure 6B, top). The difference between *E*_app_ and *E*_0_ then corresponds to the kinetic shift. Accordingly, the deduction of *P*(*E*) from the measured survival yields requires statistical‐rate theory calculations for the determination of *E*_app_ for a given experimental time scale *τ*. Despite the wide use of benzylpyridinium ions as thermometer ions, their threshold energies had not been known with certainty for quite a long time. Early quantum chemical calculations with the semiempirical AM1 method predicted dissociation energies consistent with appearance energies measured at elevated temperatures [145], but the corresponding *E*_0_ values were significantly smaller than those obtained from more recent density‐functional theory (DFT) and high‐level coupled‐cluster calculations as the example of the extensively studied *p*‐Me‐BnPy^+^ ion shows (Table 1) [140, 141, 147, 149]. Armentrout and coworkers resolved this uncertainty by providing experimental threshold energies [148], which were in good agreement with the *E*_0_ values obtained with the coupled‐cluster method (Table 1).

**TABLE 1:** *E*_0_ and *E*_app_ values for the pyridine loss from *p‐*Me‐BnPy^+^ determined by different methods. *E*_app_ values for the given *τ* were derived from *k*(*E*) curves calculated using the respective *E*_0_.

Entry	Method of *E*_0_ determination	*E*_0_, kJ mol^−1^	Method of *k*(*E*) calculations	*τ*, s	*E*_app_, kJ mol^−1^	Reference
1	AM1 calculations	154	RAC‐RRKM	1 × 10^−4^	288	[145, 146]
2	B3LYP calculations	203	RAC‐RRKM	6 × 10^−5^	444	[147]
3	CCSD(T)//B3LYP calculations	219	RAC‐RRKM	1 × 10^−4^	430^a^	[140]
4	Threshold‐CID experiments	218 ± 13	PSL‐RRKM	1 × 10^−4^	294	[148]
5	DLPNO‐CCSD(T)//PBE0‐D3 calculations	218	PSL‐RRKM	1 × 10^−4^	307	[141]

a Value read out from the corresponding *k*(*E*) curve given in ref. [140].

Relaxed surface scans by DeBord et al. and Rahrt et al. confirmed that the elimination of pyridine from the benzylpyridine ions involves a loose TS [140, 141]. To account for this feature within the RAC‐RRKM approach, several authors, such as Collette et al. [146] (Table 1, entry 1) and Naban‐Maillet et al. [147] (Table 1, entry 2), started from the reactant ion, removed the frequency of the C−N stretching vibration along the reaction coordinate, and lowered the frequencies of selected modes (see Section 2). The scaling was done in such a way that the resulting TS gave rise to an Arrhenius‐type pre‐exponential factor of *A *= 10^14^ s^−1^. This factor originates from a proposal of Vékey, who recommended values of 10^12^ ≤ *A *≤ 10^16^ s^−1^ for the modeling of reactions proceeding via loose TS [150]. In most cases, the actual RKKM calculations were performed with the MassKinetics program (see Section 3.1) and assumed experimental time scales of *τ *= 10^−4^ s (Table 1, entry 1) or slightly lower values (Table 1, entry 2). Due to the different time scales and, even more importantly, significant differences in the applied *E*_0_ values, the resulting appearance energies obtained by Collette et al. [146] and Naban‐Maillet et al. [147] differed considerably (Table 1).

DeBord et al. also used the RAC‐RRKM approach (Table 1, entry 3) but did not define the TS by scaling particular frequencies of the reactant ion [140]. Instead, they localized the TS by finding the minimum number of accessible vibrational states along the reaction coordinate *q*, *N*(*E* – *V*(*q*)), for *E *= 5.95 eV. This procedure resembles the approach of VTST (see Section 2) with the important difference that it neglected the energy dependence of the position of the TS along *q*. Given that the energy chosen for the optimization was much higher than *E*_0_, the model cannot be expected to describe the dissociation close to the threshold energy accurately. Indeed, a threshold‐CID study by Gatineau et al. showed that the TS localized by DeBord et al. for *E *= 5.95 eV was “not adequate for the lowest internal energies” [151]. Both DeBord et al. and Gatineau et al. also employed the MassKinetics software for their RRKM calculations.

In contrast to the RAC‐RRKM approach, its PSL‐RRKM counterpart is supposed to be more suitable for the modeling of loose TSs, whose interaction potential is characterized by only a relatively small anisotropy, as it should be the case for heterolytic bond cleavages, such as those in the dissociation of the BnPy^+^ ions (see Section 2). Armentrout and coworkers used PSL‐RRKM calculations for determining kinetic shifts in the analysis of the cross sections from their energy‐resolved guided‐ion beam threshold‐CID experiments (Table 1, entry 4) [148]. In doing so, they explicitly considered the *J* dependence (with a statistical distribution *P*_*E*_(*J*)) of the rate constant and assumed an interaction potential between the benzyl cation fragment ion and pyridine consisting of an ion‐dipole and an ion‐induced dipole attraction. Rahrt et al. followed the same procedure for calculating *E*_app_ values from their theoretically predicted *E*_0_ energies (Table 1, entry 5) [141]. Both Armentrout and coworkers and Rahrt et al. used the CRUNCH software (see Section 3.1) for these computations.

The obtained results provide a sound basis for assessing and comparing the performance of the different statistical‐rate theory approaches. A first indication of the validity of the PSL‐RRKM strategy is given by the excellent agreement between the *E*_0_ values for *p*‐Me‐BnPy^+^ derived from the guided‐ion beam threshold‐CID experiments and high‐level CCSD(T) calculations (Table 1, entries 3–5), which can be expected to predict correct threshold energies for the dissociation of BnPy^+^ ions. The agreement was also very good for the other benzylpyridinium ions except for *p*‐NO_2_‐BnPy^+^, which is a special case due to its more complex reactivity (see above). Had the applied kinetic model been inadequate, such close agreement could not have been achieved. As explained in Section 2, the satisfactory performance of the PSL‐RRKM approach can also be expected from fundamental considerations. For a more direct comparison between the different statistical‐rate theory variants, we focus on the calculated appearance energies. Obviously, no straightforward comparison is possible for calculations that used significantly different *E*_0_ values as input or determined *E*_app_ for different time scales *τ*. Therefore, we exclude the results by Collette et al. [146] (Table 1, entry 1) and Naban‐Maillet et al. [147] (Table 1, entry 2) from our analysis because they are based on an exceptionally low threshold energy and a deviating time scale, respectively. In contrast, the calculations performed by DeBord et al. [140] (Table 1, entry 3), Armentrout and coworkers [148] (Table 1, entry 4), and Rahrt et al. [141] (Table 1, entry 5) all started from very similar conditions and, thus, lend themselves to a direct comparison. The two studies relying on the PSL‐RRKM approach afforded quite similar appearance energies of 294 and 307 kJ mol^−1^ (Table 1, entries 4 and 5). Given that both investigations used identical *E*_0_ values and the same statistical‐rate theory model, one might have expected an even closer agreement of their predictions. Possibly, the small remaining difference in the *E*_app_ values originates from differences in the calculated vibrational frequencies of the TS and reactant (B3LYP vs PBE0‐D3 frequencies), which were applied for computing their sum and density of states, respectively (see Section 2). In comparison to the appearance energies derived from the PSL‐RRKM calculations, that obtained from the RAC‐RRKM calculation, *E*_app_ = 430 kJ mol^−1^ (Table 1, entry 3), is much higher, corresponding to a significantly larger kinetic shift. Differences in the computed kinetic shifts result in the derivation of different *P*(*E*) distributions, as the studies of Naban‐Maillet et al. and Armentrout and coworkers illustrate. The former, deriving a large kinetic shift from RAC‐RRKM calculations, determined an effective temperature for ions produced by ESI of *T*_ESI_ = 1620 K [147], whereas the latter, deriving a much smaller kinetic shift from PSL‐RRKM calculations, arrived at a temperature of *T*_ESI_ = 725 ± 23 K [148]. Although no direct comparison between these values is feasible due to differences in the experimental details, it appears obvious that the large deviation between the two temperatures reflects shortcomings in one of the theoretical methods used.

From the discussion above, it is clear that we consider the aforementioned RAC‐RRKM calculations insufficient. That being said, the RAC‐RRKM approach itself is not necessarily incapable of modeling the dissociation of the BnPy^+^ ions correctly. Exploratory calculations based on a vibrational‐frequency scaling corresponding to an Arrhenius‐type pre‐exponential factor of *A *= 10^18^ s^−1^ instead of 10^14^ s^−1^ could successfully reproduce the results of SSACM computations for the dissociation of *p*‐H‐BnPy^+^ [152]. Likewise, defining the TS by finding the minimum of *N*(*E* – *V*(*q*)) for an energy smaller than *E *= 5.95 eV would be more suitable for describing the dissociation behavior close to the threshold energy or determining appearance energies for the relevant time scales. At lower energies, the TS moves further outward along *q* and thereby becomes less tight. Although a properly chosen RAC‐RRKM method, thus, is able to model correctly the dissociation of BnPy^+^ ions and other reactions proceeding via loose TSs at least for a certain energy range, it does not provide a straightforward means for identifying the appropriate TS. In this respect, the PSL‐RRKM approach is superior and appears well‐suited for describing heterolytic bond cleavages, whose loose TSs exhibit interaction potentials with only relatively small anisotropic contributions.

Further studies investigated the suitability of related systems as thermometer ions and, in doing so, conducted statistical‐rate theory calculations for the quantitative analysis of measured survival yields. Rahrt et al. probed benzhydrylpyridinium ions and again employed the PSL‐RRKM approach for the calculation of appearance energies of dissociation as well as for fitting energy‐resolved guided‐ion beam threshold‐CID data (using the CRUNCH program) [141]. The very good agreement between the derived *E*_0_ values and those computed by high‐level coupled‐cluster calculations suggests that the PSL‐RRKM calculations correctly modeled the dissociation behavior. Asakawa and Saikusa [153] introduced polyfluorinated benzylpyridinium ions. For the analysis of the survival yields, they applied the RAC‐RRKM model (using the MassKinetics program) but scaled the frequencies of selected modes in such a way that the resulting TS gave rise to an Arrhenius‐type pre‐exponential factor of *A *= 10^20^ s^−1^ instead of 10^14^ s^−1^. With this method, they determined significantly smaller kinetic shifts for the dissociation of several BnPy^+^ ions than the previous RAC‐RRKM calculations and, thus, obtained results in line with those from the PSL‐RRKM calculations (a direct comparison is prevented by deviating experimental time scales).

### 4.3 Dissociation of Organometallic Anions

Besides purely organic ions, organometallic ions have been among the most extensively studied systems in gas‐phase ion chemistry [154]. Presumably, this special interest reflects the versatile reactivity of this class of compounds as well as the difficulty in characterizing their behavior in solution, where the operation of dynamic equilibria typically severely complicates the analysis [155]. Several studies employed sophisticated instrumentation for determining energy‐resolved reaction cross sections and also made use of statistical‐rate theory calculations for fitting the measured data [33, 156, 157, 158]. Other investigations with commercial equipment remained at the qualitative or semiquantitative level but also included quantum chemical calculations for aiding in the interpretation of the experimental results by computing relevant potential energy surfaces [159, 160, 161, 162]. In most cases, the experiments measured relative signal intensities of the precursor and fragment ions (typically under variation of the kinetic energy *E*_LAB_). As the observed fragmentation reactions proceed irreversibly, the obtained relative signal intensities of the fragment ions correspond to relative rate constants. Obviously, no direct comparison between the measured rate constants and the calculated PES is feasible, thus limiting the value of the quantum chemical calculations.

**FIGURE 7:**
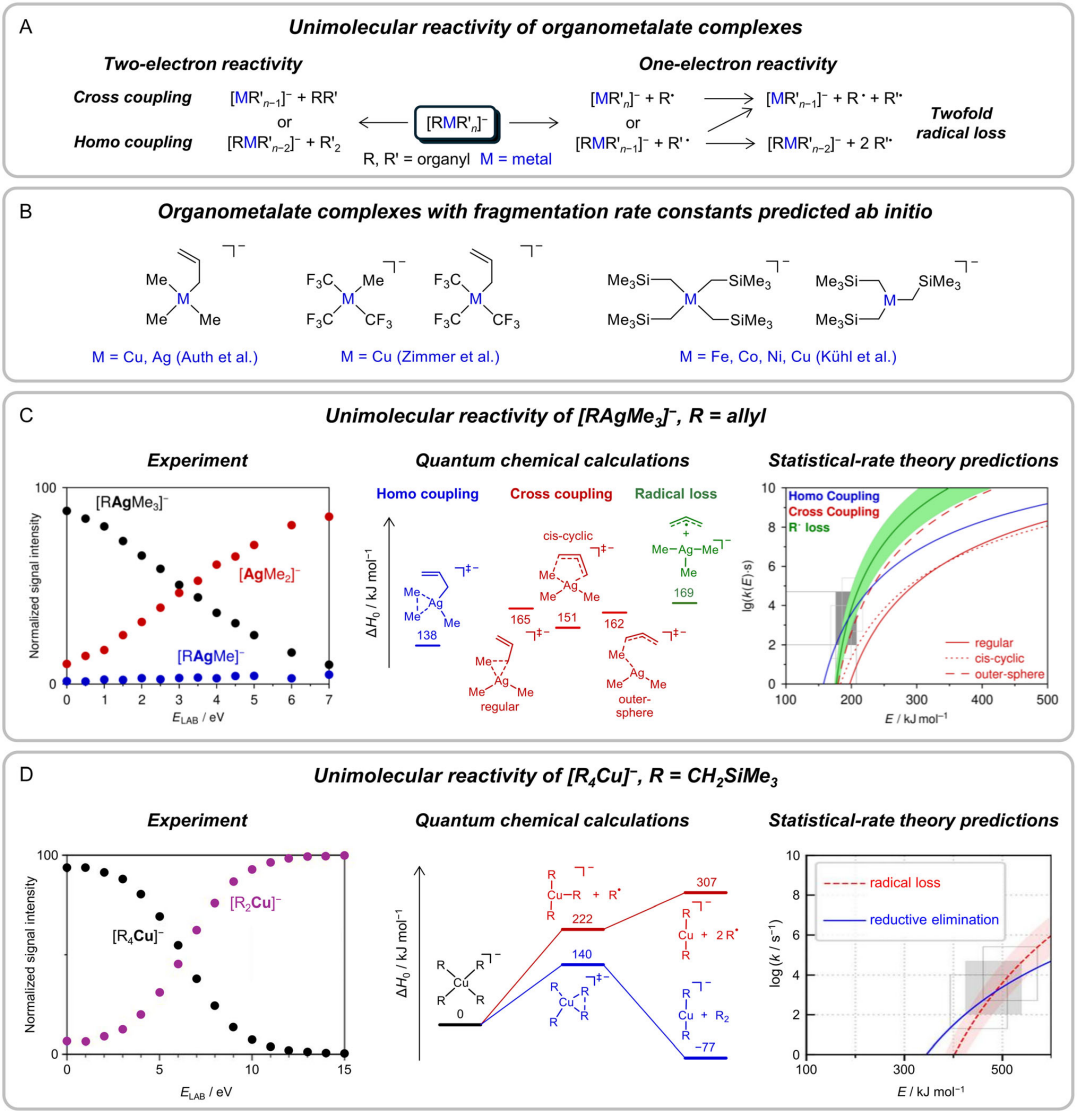
Gas‐phase fragmentation of organometallic complexes, for which rate constants have been predicted ab initio by means of statistical rate theory. (A) Two‐ and one‐electron type dissociation reactions of [RMR′_*n*_]^−^ complexes. (B) Probed complexes, with the ground‐state structure (quadratic planar vs tetrahedral or trigonal vs T‐shaped) depending on the metal M. (C) Fragmentation of [RAgMe_3_]^−^, R = allyl [163]. (Left) Normalized signal intensities of [RAgMe_3_]^−^ and fragment ions formed upon collision‐induced dissociation at different energies. (Middle) Relative enthalpies of TS structures obtained from DLPNO‐CCSD(T)//PBE‐D3 (homo coupling, regular cross coupling, radical loss) and CASPT2//PBE‐D3 calculations (*cis*‐cyclic and outer‐sphere cross coupling). (Right) Reaction rate constants *k*(*E*) obtained from statistical‐rate theory computations (RAC‐RRKM: homo and cross coupling, SSACM: radical loss) based on the results from the quantum chemical calculations. The green shaded area indicates the uncertainty of rate constants of the radical‐loss channel resulting from the assumed rigidity factor of log *f*_rigid_ = −2 ± 1. The filled (open) gray box(es) indicate the relevant *k*(*E*) range(s) for an estimated experimental time scale of *τ *= 10^−4^ s (*τ* = 2 × 10^−5^ and 5 × 10^−4^ s). (D) Fragmentation of [R_4_Cu]^−^, R = CH_2_SiMe_3_ [164]. (Left) Normalized signal intensities of [R_4_Cu]^−^ and fragment ions formed upon collision‐induced dissociation at different energies. (Middle) Energy diagram for the unimolecular reactivity (reductive elimination vs twofold radical loss) obtained from DLPNO‐CCSD(T)//ωB97X‐D calculations. (Right) Reaction rate constants *k*(*E*) obtained from statistical‐rate theory computations (RAC‐RRKM: reductive elimination, SSACM: radical loss) based on the results from the quantum chemical calculations. The red shaded area indicates the uncertainty of rate constants of the radical‐loss channel resulting from the assumed log *f*_rigid_ = −2 ± 1. The filled (open) gray box(es) indicate the relevant *k*(*E*) range(s) for an estimated *τ *= 10^−4^ s (*τ *= 2 × 10^−5^ and 5 × 10^−4^ s). Adapted with permission from refs. [163] and [164]. Copyright 2021 and 2025, Wiley‐VCH.

Of course, this problem can be overcome if the results of the quantum chemical calculations are combined with statistical‐rate theory computations. This approach has recently been pursued for the analysis of the gas‐phase fragmentation of organometalate complexes [RMR′_*n*_]^−^, R, R′ = organyl, M = metal (Figure 7) [163, 164, 165]. Here, one of the key questions is whether these complexes react via a concerted reductive elimination of the coupling product, i.e., a typical two‐electron reaction, or via the consecutive loss of two organyl radicals, i.e., typical one‐electron reactions (Figure 7A). Experimentally, the distinction between these two different pathways can be difficult if the activation energy for the second radical loss is low, and this reaction proceeds fast. In this case, the lifetime of the intermediate [MR′_*n*_]^−^ or [RMR′_*n*−1_]^−^ complex may be so short that it escapes detection. Therefore, the failure to observe such an intermediate does not necessarily exclude the operation of a twofold radical loss. For heteroleptic complexes, a second question concerns the chemoselectivity of the reaction (Figure 7A). The fragmentation process can either involve two different organyl groups or two identical ones. If the fragmentation occurs in a concerted manner and results in the coupling of the two organyl groups, these two different reactions are usually referred to as cross and homo coupling (in the condensed phase, coupling products can also form via the twofold radical pathway if the released radicals are trapped in the solvent cage sufficiently long to enable their recombination). In synthetic applications, only the cross‐coupling product is desired.

A first example is given by the dissociations of [RCuMe_3_]^−^ and [RAgMe_3_]^−^, R = allyl (Figure 7B and C) [163]. In earlier experiments with a quadrupole‐ion trap, Putau et al. had found the gas‐phase fragmentation of the former ion to afford mainly [RCuMe]^−^ along with smaller amounts of [CuMe_2_]^−^ and assigned these fragment ions to the products of concerted reductive eliminations [161]. In later experiments with a quadrupole time‐of‐flight hybrid instrument, Auth et al. confirmed the formation of [RCuMe]^−^ and [CuMe_2_]^−^, but in addition also observed small quantities of [CuMe_3_]^−^ [163]. The latter clearly showed the occurrence of a one‐electron reaction. CID of [RAgMe_3_]^−^ only afforded [RAgMe]^−^ and [AgMe_2_]^−^ [163, 166]. In contrast to the fragmentation of the cuprate, in this case, the homoleptic fragment ion [AgMe_2_]^−^ prevailed over its heteroleptic counterpart [RAgMe]^−^. Quantum chemical calculations revealed the significant complexity of these systems. Due to the special nature of the allyl substituent and its ability to form a new bond both via its α and γ sites, several different mechanistic pathways leading to the cross‐coupling product exist (Figure 7C) [163]. Some of these pathways involve TSs, whose wave functions have a considerable multiconfigurational character and therefore require special methods in their theoretical description. As the first step toward predicting the relative reactivities, the activation energies of the different reaction channels were computed. For the homo‐coupling and the cross‐coupling pathways, this approach is conceptually straightforward as these reactions proceed via well‐defined TSs. The situation is different for the primary radical expulsions, i.e., the first steps of the twofold radical losses, which compete with the homo‐ and cross‐coupling channels. These reactions correspond to direct bond cleavages and therefore were assumed to be barrierless (cf. Section 4.1). As the primary radical losses are associated with much higher bond dissociation energies (*D*_0_(Me_3_Cu^−^−R) = 177, *D*_0_(Me_3_Ag^−^−R) = 169 kJ mol^−1^) than the secondary ones (*D*_0_(Me_2_Cu^−^−Me) = 33, *D*_0_(Me_2_Ag^−^−Me) = 7 kJ mol^−1^), they correspond to the rate‐limiting steps of the overall fragmentations [163].

Importantly, the TSs of the different pathways differ in their rigidity. While those of the homo‐coupling as well as the regular and the *cis*‐cyclic cross‐coupling channels are typical tight ones, the TSs of the outer‐sphere cross‐coupling pathways have much looser characters because they no longer fully bind the allyl radical, which therefore is able to move about quite freely [167]. Obviously, the ability of the allyl radical and the metal fragments to move freely is even higher in the dissociation limits of the radical‐loss pathways, which therefore achieve the maximum degree of looseness of all TSs identified for the fragmentation of [RCuMe_3_]^−^ and [RAgMe_3_]^−^. Thus, the unimolecular reactivity of the [RCuMe_3_]^−^ and [RAgMe_3_]^−^ complexes appears to be a typical case, in which no meaningful comparison of the relative facility of the different dissociation channels is possible without the explicit consideration of the energy dependence of the rate constants. Such an explicit consideration requires statistical‐rate theory calculations.

In this specific case, Auth et al. used the VariFlex program (see Section 3.2) for the calculations [163]. Exploratory computations found the influence of rotational excitation to be low, for which reason the *J* dependence of the rate constants was neglected, and the *k*(*E*,*J*) values were approximated as *k*(*E*,*J* = 0). For the homo‐coupling and cross‐coupling reaction channels, the RAC‐RRKM model was applied, and the calculated Δ*H*_0_^‡^ energy barriers were used for the TS energies *E*_0_. For the radical‐dissociation pathways, Auth et al. calculated rate constants by PST and employed the bond‐dissociation energies *D*_0_ as activation energies *E*_0_. Moreover, for all dissociation channels, Auth et al. considered reaction‐path degeneracies *σ* by analyzing the geometries and taking into account spin multiplicities of the involved molecular configurations. To correct for the likely shortcomings of the PST approach with respect to the treatment of barrierless homolytic bond cleavages, Auth et al. multiplied the obtained rate constants by an energy‐independent rigidity factor, in line with the SSACM method [163]. In their study, Auth et al. directly referred to the work of Halbert and Bouchoux, who had demonstrated the feasibility of this procedure for the dissociation of the *n*‐butylbenzene radical cation (see section 4.1) [136]. In fact, Auth et al. applied the rigidity factor determined for the butylbenzene system (*f*_rigid_ = 10^−2^) to the dissociation of the [RCuMe_3_]^−^ and [RAgMe_3_]^−^ complexes. This procedure appears justified given the related reaction mechanisms of the probed systems, the similar sizes and *E*_0_ values, as well as the comparable energy regimes of the performed experiments. Nevertheless, Auth et al. gave conservative error bars of log (Δ*k*(*E*)/*k*(*E*)) = ±1 to account for the presumably imperfect accuracy of the results predicted for the radical‐dissociation channel.

The calculation of the theoretical *k*(*E*) curves as such was not yet sufficient for a comparison with the experimental findings because the energy range sampled in the latter was unknown at the outset. The use of a simple commercial instrument for the performed experiments means that the obtained energy distributions were not well defined. Furthermore, the number of collisions in the experiments was not well defined either and exceeded the single‐collision limit, thereby preventing a straightforward conversion of the energies from the laboratory into the center‐of‐mass frame. Instead, Auth et al. estimated the effective energy range probed in the experiments from the approximate time scale of the latter [163]. Arguing that well‐observable fragmentation reactions should involve fragmentation yields between 1% and 99%, they determined the rate constants corresponding to these limits based on an assumed effective time scale of *τ *≈ 10^−4^ s for the employed quadrupole‐TOF (time‐of‐flight) instrument. They went on to map these limiting rate constants on the predicted *k*(*E*) curves. For [RAgMe_3_]^−^, they arrived at energies of up to approx. 200 kJ mol^−1^ (∼2 eV) deposited into the precursor ions while the experiments were performed at nominal energies of 0 ≤ *E*_LAB_ ≤ 7 eV. With this information, Auth et al. could localize the experimentally accessed window within the *k*, *E* space and compare it with the *k*(*E*) curves predicted ab initio. In the case of [RCuMe_3_]^−^, this comparison showed that the homo‐coupling channel should be the preferred pathway within the experimental energy range, in full accordance with the experimental findings. At the upper end of the accessible energy range, the radical dissociation was predicted to start competing, which was consistent with the observation of small amounts of [Me_3_Cu]^−^ upon CID in the quadrupole‐TOF instrument.

For the fragmentation of [RAgMe_3_]^−^, the computed *k*(*E*) curves for the experimentally accessible energy range indicated that at lower collision energies, homo‐coupling would be the preferential dissociation channel [163]. However, at higher energies, it should be outcompeted by the radical dissociation. The primary fragment ion [Me_3_Ag]^−^ resulting from this reaction was predicted to be hardly stable toward the loss of a methyl radical, which explains why it was not detected in the experiments. According to the calculations, not only the radical dissociation, but also the outer‐sphere cross‐coupling pathway contributed to the formation of [Me_2_Ag]^−^ in the quadrupole‐TOF experiments, thereby rationalizing its predominance over the homo‐coupling fragment ion [RAgMe]^−^. Interestingly, the outer‐sphere cross‐coupling channel was found to contribute mainly at higher energies. As discussed above, this behavior reflects the partial release of the allyl radical in the TS and a loose character of the latter. Hence, the main reason for the deviating reactivity of the argentate complex compared to its copper congener is the former's higher propensity for radical‐type reactions. Clearly, the analysis by statistical‐rate theory calculations proved instrumental in the interpretation of the experimental results and differentiating between one‐ and two‐electron processes. As Auth et al. pointed out, the unraveled distinct gas‐phase reactivities of [RCuMe_3_]^−^ and [RAgMe_3_]^−^ even helped to understand previously reported mechanistic differences between copper‐ and silver‐mediated allylation reactions in solution [163].

The methodology established by Auth et al. was also applied to the analysis of related systems. First, Zimmer et al. employed essentially the same approach (including the use of a rigidity factor of *f*_rigid_ = 10^−2^) to predict rate constants for the dissociation of [(CF_3_)_3_CuMe]^−^ and [(CF_3_)_3_CuR]^−^, R = allyl [165]. Obviously, these species closely resemble the [RCuMe_3_]^−^ complex studied by Auth et al. When subjected to CID in a quadrupole‐ion trap, both [(CF_3_)_3_CuMe]^−^ and [(CF_3_)_3_CuR]^−^ afforded [(CF_3_)_3_Cu]^−^ and [(CF_3_)_2_Cu]^−^ as fragment ions, although in different relative abundances. The former predominated for the fragmentation of [(CF_3_)_3_CuR]^−^, but only reached low signal intensities for the dissociation of [(CF_3_)_3_CuMe]^−^. While it is obvious that [(CF_3_)_3_Cu]^−^ corresponded to a primary fragment ion resulting from the loss of the allyl radical, [(CF_3_)_2_Cu]^−^ could be a secondary fragment ion originating from [(CF_3_)_3_Cu]^−^ by the consecutive release of a CF_3_^•^ radical or another primary fragment ion formed by the direct release of the CF_3_Me or CF_3_R coupling product from the respective precursor ion. Thus, the unimolecular chemistry of [(CF_3_)_3_CuMe]^−^ and [(CF_3_)_3_CuR]^−^ again is governed by the competition between one‐ and two‐electron reactions. Remarkably, both competing [(CF_3_)_2_Cu]^−^ formation channels could be distinguished experimentally by the spontaneous consecutive elimination of CF_2_, which only occurred in the case of a coupling reaction [165]. In this case, the high exothermicity of the primary reductive elimination resulted in sufficiently high amounts of internal energy of [(CF_3_)_2_Cu]^−^ to drive the consecutive loss of CF_2_. For [(CF_3_)_3_CuMe]^−^, the consecutive liberation of CF_2_ proceeded to a significant extent and, thus, pointed to the occurrence of the concerted reductive elimination of CF_3_Me as primary reaction. In contrast, the complete absence of the CF_2_‐elimination product ion in the case of [(CF_3_)_3_CuR]^−^ showed that here the [(CF_3_)_2_Cu]^−^ fragment ion exclusively originated from the twofold radical loss.

The statistical‐rate theory calculations based on the results from quantum chemical computations fully confirmed these findings [165]. According to the calculations for [(CF_3_)_3_CuMe]^−^, the concerted reductive elimination of CF_3_Me predominated over the whole experimental energy range, whereas the loss of a methyl radical only competed at the higher accessible energies. For [(CF_3_)_3_CuR]^−^, only the loss of an allyl radical was computed to be feasible under the experimental conditions. These predictions were based on an estimated experimental time scale of *τ *≈ 10^−2^ s for the quadrupole‐ion trap. This value is significantly higher than that assumed for the quadrupole‐TOF instrument used for the fragmentation experiments on [RCuMe_3_]^−^ and [RAgMe_3_]^−^ (see above) and points to the different operation principles of the two apparatus. Again, the statistical‐rate theory calculations helped in the mechanistic analysis and elucidation of radical dissociations, highlighting these as feasible pathways for high‐valent trifluoromethylated cuprates. Meanwhile, the operation of such mechanisms could also be confirmed for analogous reactions in solution [168].

A further system is given by the fragmentation of the homoleptic organometalates [R_*n*_M]^−^, R = CH_2_SiMe_3_, *n *= 3 and 4, M = Fe, Co, Ni, and Cu (Figure 7D) [164]. In the present context, the dissociation of the tetra‐coordinated complexes [R_4_M]^−^ is of particular interest because the reactivity of these species is again characterized by the competition between concerted reductive eliminations, i.e., two‐electron processes, and radical losses, i.e., one‐electron processes. Using a quadrupole‐TOF instrument, Kühl et al. observed that CID of the ferrate and cobaltate complexes resulted exclusively in the radical dissociation. For the nickelate, the analogous reaction also predominated at lower collision energies. At medium energies, Kühl et al. found evidence for the occurrence of a concerted reductive elimination as a minor channel. The cuprate, in turn, did not show any loss of the R^•^ radical upon CID but only afforded [R_2_Cu]^−^, which apparently pointed to the exclusive operation of a concerted reductive elimination. The calculated energy profiles for the two reaction types also seemed to be consistent with this assessment. For the ferrate and cobaltate, the computed barriers associated with the concerted reductive elimination lay above the energy required for the homolytic cleavage of the M−R bond. In the case of the nickelate, both pathways had very similar energetic requirements. Given that the tightness of the TS of the concerted reductive elimination disfavors this reaction relative to the radical dissociation, the latter should predominate, as observed experimentally. For the cuprate, the barrier of the reductive elimination was calculated to be more than 80 kJ mol^−1^ lower than the energy necessary for the radical loss, which appears to be consistent with a strong preference for the former channel (Figure 7D).

Using the results of the quantum chemical computations as input, Kühl et al. went on to perform statistical‐rate theory calculations [164]. In doing so, they again adopted the methodology established by Auth et al. (see above) [163], but employed the MESS software (see Section 3.2) for the actual calculations. Following the procedure by Auth et al., they corrected the rate constants determined by PST for the radical dissociations by a rigidity factor of *f*_rigid_ = 10^−2^ (SSACM approach). For estimating the experimentally relevant rate constant ranges, an instrumental time scale of *τ *= 10^−4^ s was assumed, like in the study of Auth et al., who had used the very same instrument [163]. In this way, Kühl et al. obtained rate constants in line with the preliminary interpretation of the experimental results for the ferrate, cobaltate, and nickelate complexes based on their quantum chemical calculations. In contrast to the qualitative analysis, the predicted rate constants for the cuprate indicated that the radical dissociation competes effectively with the reductive elimination. In fact, the former was calculated to prevail over the latter for the upper half of the experimentally accessible energy range (Figure 7D). In comparison to the [RCuMe_3_]^−^ complex, R = allyl, studied by Auth et al. [163], the [R_4_Cu]^−^ ion, R = CH_2_SiMe_3_, is larger and has a higher Cu—R bond‐dissociation energy. Accordingly, the efficient fragmentation of the latter required significantly higher energies, which may explain why the resulting [R_3_Cu]^−^ primary fragment ions underwent an instantaneous secondary dissociation to afford [R_2_Cu]^−^, despite the considerable activation energy of 85 kJ mol^−1^ of this step (Figure 7D). Thus, the work of Kühl et al. demonstrated that the analysis of the PESs alone is insufficient for predicting the reactivity even at a qualitative level, but that explicit statistical‐rate theory calculations are indispensable. With the available software and modern quantum chemical methods, relatively large systems can be treated in an efficient manner and with apparently good success. However, a rigorous assessment of the performance of the applied methodology is not possible unless high‐quality experimental data are provided for comparison. As discussed above, such data typically cannot be obtained with commercial instruments. Nonetheless, even for these instruments, efforts can and should be taken toward a better characterization of the experimental conditions, an improved knowledge of which would allow for a more targeted and, thus, more accurate theoretical modeling. For CID experiments, as those presented here, a more accurate determination of the energy distribution of the reactant ion, as well as of the instrumental time scale, appears particularly desirable.

### 4.4 Protonation of Organometallic Ions

Besides the unimolecular reactivity of organometallic ions in the gas phase, the bimolecular reactivity of these species has been investigated to gain detailed insight into metal‐mediated processes. Typically, the presence of a reaction partner raises the complexity of the system considerably. For this reason, the mechanistic analysis of bimolecular gas‐phase reactions of organometallic ions can be quite challenging. The safest way to test the validity of a given mechanistic hypothesis, thus, appears to be the combination of quantum chemical calculations with statistical‐rate theory/master‐equation computations to predict rate constants for a direct comparison with the results from kinetic measurements. While this approach is not limited to a specific system, a great deal of attention has been paid to protonation processes [169, 170, 171, 172, 173]. First, proton‐transfer processes correspond to the simplest and most fundamental of all reactions and, thus, lend themselves particularly well to an in‐depth mechanistic analysis. Second, typical organometallic complexes react as bases and therefore can be readily protonated. In fact, protodemetalation constitutes one of the most important elementary steps of organometallics [174, 175, 176].

Ion‐molecule reactions of mass‐selected ions can be easily probed with commercial instruments if the latter permit the controlled infusion of the neutral substrate or are modified for this purpose. In the present context, the main interest lies in the determination of rate constants, which can be achieved by the measurement of ion intensity profiles as a function of space or time. The latter variant requires the storage of ions for variable times, which can be accomplished with Fourier‐transform ion‐cyclotron resonance or quadrupole‐ion trap instruments. While earlier studies applied almost exclusively the former to this purpose, the latter have been used more often recently [7, 23]. These instruments are operated with helium as a bath gas (*p *≈ 10^−3^ mbar) for collisional thermalization of the ions. Several studies have confirmed that under typical conditions, ion thermalization is efficient and results in ion temperatures at or slightly above room temperature [92, 93, 94]. The presence of thermal ion distributions is of crucial importance because it ensures well‐defined experimental conditions and thereby greatly facilitates a direct comparison of the measured rate constants with those calculated by statistical rate theory.

The theoretical prediction of rate constants of ion‐molecule reactions occurring in a quadrupole‐ion trap involves a significantly higher degree of complexity than those of unimolecular dissociations. Besides the collisions between the ion and the neutral substrate, those between energized ionic reaction intermediates and the helium background gas have to be considered because they affect the internal energy distributions of the former. Furthermore, the potential energy surfaces of ion‐molecule reactions are typically inherently more complex than those of dissociation processes. At the same time, ion‐molecule reactions offer the great advantage that their measured rate constants can be directly compared with the theoretical predictions and, thus, allow for a rigorous quantitative assessment of the accuracy of the latter.

**FIGURE 8:**
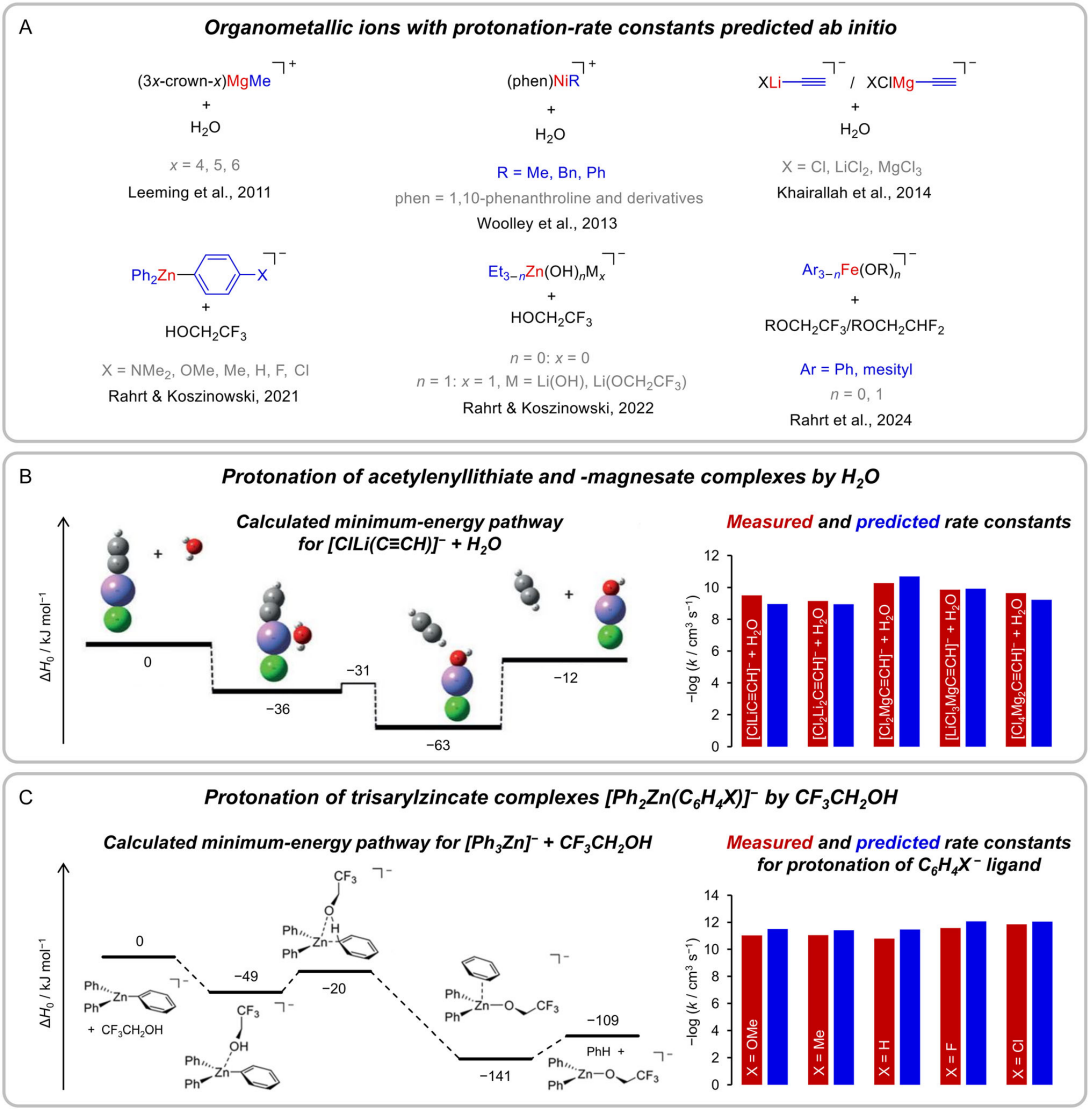
Gas‐phase protonation reactions of organometallic complexes, for which rate constants have been predicted ab initio by means of statistical rate theory. (A) Overview of probed systems [177, 178, 179, 180, 181, 182]. (B) Protonation of acetylenyllithiate and ‐magnesate complexes by H_2_O [179]. (Left) Minimum‐energy pathway for the reaction of [ClLi(C≡CH)]^−^ obtained from G3SX calculations. (Right) Comparison of measured (red) and theoretically predicted (blue) negative logarithmic rate constants from mass‐spectrometric experiments and statistical‐rate theory/master‐equation calculations (employing the G3SX results), respectively. (C) Protonation of heteroleptic trisarylzincate complexes [Ph_2_Zn(*p*‐C_6_H_4_‐X)]^−^ by CF_3_CH_2_OH, X = NMe_2_, OMe, Me, H, F, Cl [180]. (Left) Minimum‐energy pathway for the reaction of [Ph_3_Zn]^−^ obtained from DLPNO‐CCSD(T)//ωB97X‐D3 calculations. (Right) Comparison of measured (red) and theoretically predicted (blue) negative logarithmic rate constants for the protonation of the *p*‐C_6_H_4_‐X^−^ carbanionic ligand from mass‐spectrometric experiments and statistical‐rate theory/master‐equation calculations (employing the DLPNO‐CCSD(T)//ωB97X‐D3 results), respectively. Adapted with permission from refs. [179] and [180]. Copyright 2014 and 2021, Wiley‐VCH and American Chemical Society.

The first systems considered here correspond to hydrolysis reactions of ligated organomagnesium and nickel cations, as well as organolithiate and magnesate anions investigated by O’Hair and coworkers (Figure 8A) [177, 178, 179]. Despite the heterogeneity of these ions, they were all found to react with water in an analogous fashion and abstract a proton from the latter (besides minor other competing reactions in some cases). The proton binds to the carbanionic organyl ligand R^−^, which is released as neutral hydrocarbon RH, whereas the remaining hydroxide OH^−^ coordinates to the metal center. In all cases, O’Hair and coworkers determined the rate constants of the hydrolysis reactions by kinetic measurements carried out in a quadrupole‐ion trap under pseudo‐first‐order conditions with water being present in large excess relative to the reactant ions. Logarithmic plots of the ion‐intensity time profiles showed good linearity, demonstrating the effectiveness and reliability of this approach. Nonetheless, the uncertainties of the derived absolute rate constants were relatively high (30%) due to the difficulties in the accurate determination of the absolute partial pressure of water in the experiments [177, 178, 179].

Quantum chemical calculations on the studied systems were performed with standard DFT methods (B3LYP or M06) [177, 178] or the high‐level G3SX composite approach [179]. The obtained PESs corresponded to simple double‐well potentials, which feature reactant ion‐molecule complexes, TSs, and product ion‐molecule complexes as stationary points connecting reactants and products, or more complex variants thereof (cf. Figure 8B for the hydrolysis of [ClLi(C≡CH)]^−^). All the systems have closed electron shells, for which reason the results from the quantum chemical calculations are expected to be fairly reliable, especially those obtained with the G3SX method.

For the kinetic simulations, O’Hair and coworkers made use of different versions of the MultiWell program suite (see Section 3.2). Microcanonical rate constants *k*(*E*) for elementary steps associated with well‐defined TSs, such as the actual proton‐transfer reactions from water onto the carbanionic ligands, were calculated with the help of standard RAC‐RRKM theory. To this end, the energies, structures, and vibrational frequencies obtained from the quantum chemical calculations were employed as input to compute densities and sums of states as well as moments of inertia. As explained in Section 2, this approach is not appropriate for barrierless elementary reactions. Elementary reactions of this type are the initial formation of ion‐molecule complexes between the organometallic ion and H_2_O, as well as the release of the newly formed hydrocarbon RH from the metal‐hydroxide complex. The formation of the ion‐molecule complexes was supposed to proceed at the collision rate. As detailed in Section 2, the inverse Laplace transform method can be used to obtain microcanonical rate constants for the complex formation from their canonical counterparts, which O’Hair and coworkers calculated with the ADO theory [179]. Moreover, by making use of the corresponding equilibrium constant predicted by the quantum chemical computations, the canonical rate constant for the RH complex formation can be converted into microcanonical reaction rate constants for the dissociation of the latter with the help of the inverse Laplace transform method.

The thus obtained *k*(*E*) functions for the individual elementary steps of a given reaction mechanism then served as input for stochastic master‐equation simulations [177, 178, 179]. Importantly, these simulations accounted for collisional energy transfer between the energized ionic reaction intermediates and the surrounding helium bath in the quadrupole‐ion trap. To model this energy transfer as accurately as possible, both the pressure and the temperature of the helium had to be known. In all cases, O’Hair and coworkers assumed a temperature of *T *= 298 K, whereas they used slightly deviating pressures in their different studies (*p *= 1.75, 1.752, and 2.0 mtorr), possibly reflecting small changes in the experimental conditions. Woolley et al. modeled the collisions between the ionic intermediates and the helium atoms according to the Langevin collision rate [178]. In contrast, Khairallah et al. assumed the collisions to proceed according to the Lennard‐Jones interaction potential [179, 183]. The latter choice appears less appropriate because the Lennard‐Jones potential describes the interaction between neutrals, which is significantly weaker than that between ions and neutrals and, thus, can be expected to underestimate the true number of collisions.

To account for the effect of a collision between an ionic intermediate and a helium atom, transition probabilities for the conversion of the ion into another energetic state must be known. In line with previous work on the collisional energy transfer of neutral systems in the gas phase, O’Hair and coworkers only considered deactivating collisions, in which the energy of the ionic intermediate is reduced from *E* to *E*′ in a single collision event. The probability for such a transition at a helium‐bath temperature *T* is then given as *p*(*T*, *E*′, *E*). An expression for *p*(*T*, *E*′, *E*) derived from first principles is unknown, for which reason it must be approximated empirically. Most commonly, a so‐called mono‐exponential down model is used, in which the transition probability decreases for higher amounts of lost energy Δ*E*_down_ = *E* − *E*′ [184, 185]. Typically, a specific form of the model applied is not characterized by *α*(*T*, *E*) itself but by giving the average energy transferred in a collision. For the deactivation of small organic molecules, average deactivation energies of ⟨Δ*E*⟩_down_ = 200 cm^−1^ (corresponding to approx. 2.4 kJ mol^−1^) are generally assumed. Khairallah et al. also used this value for modeling the collisional energy transfer in the hydrolysis of acetylide lithiates and magnesates [179]. In contrast, Woolley et al. employed different average deactivation energies for the ionic intermediates of the hydrolysis of (phen)NiMe^+^ and (phen)NiPh^+^ (150 ≤ ⟨Δ*E*⟩_down_ ≤ 500 cm^−1^, phen = 1,10‐phenanthroline and derivatives thereof) to optimize the agreement between the observed and the calculated reactivity [178]. Thus, this study did not achieve truly ab initio predictions of rate constants.

A comparison of the measured and computed hydrolysis rate constants of the different systems investigated by O’Hair and coworkers showed that in all cases but one, the theoretical calculations predicted the correct order of magnitude [177, 178, 179]. The exception was the hydrolysis of [(15‐crown‐5)MgMe]^+^, for which the calculations drastically underestimated the rate constant (*k*_theo_ = 1.72 × 10^−16^ vs *k*_exp_ = 3.97 × 10^−12^ cm^3^ s^−1^) [177]. The authors attributed this failure to the prediction of the TS of the proton‐transfer step lying 13 kJ mol^−1^ above the entrance channel. Adjusting this energy to −7 kJ mol^−1^ made the calculated rate constant match the experimental value [177]. While this finding possibly pointed to a limited accuracy of the quantum chemical calculations, it could also reflect the occurrence of an alternative reaction pathway not included in the theoretical analysis. Given the considerable complexity of this system, an exhaustive search of all conceivable mechanistic pathways is notoriously difficult. For the reactions of the organonickel cations as well as for the acetylenyllithiates and ‐magnesates, the calculated rate constants deviated from the experimental ones by a factor of ≲ 2 (Figure 8B for the hydrolysis reactions of the latter). This good agreement does not necessarily imply a high accuracy of the results of the underlying quantum chemical calculations. For all of these systems, the TSs of the proton‐transfer steps lie energetically significantly below the entrance channel (cf. Figure 8B for the hydrolysis of [ClLi(C≡CH)]^−^) [178, 179]. Accordingly, they do not exert severe kinetic restraints on the overall reactions, which therefore proceed with relatively high efficiencies (defined as the ratios of *k* and the collision rate). For the accurate modeling of such reactions, the correct description of the capture process may actually be more important than that of the remaining steps. A different situation is to be expected for reactions involving tight TSs whose energies do not lie significantly below the entrance channel. In these cases, the predicted rate constants will depend strongly on the activation barriers and the employed kinetic model. In addition, corrections for hindered internal rotations [51], anharmonicities of vibrations [58], and tunneling effects [103, 186, 187, 188] supposedly can improve the accuracy of the predicted rate constants for reactions with significant kinetic constraints.

Further protonation reactions of organometallic ions were investigated by Rahrt et al., who also used a quadrupole‐ion trap for the measurement of absolute rate constants (Figure 8A). Two of these studies probed the protolysis of organozincate complexes by 2,2,2‐trifluoroethanol, which is a stronger acid than water, both in the gas phase and in solution [180, 181]. In analogy to the proton‐transfer processes explored by O’Hair and coworkers, these reactions brought about the protonation of the zinc‐bound carbanionic (or hydroxo) ligands, which were released as the corresponding hydrocarbons (or water). The resulting alkoxide simultaneously formed a new bond to the zinc center. Both studies focused on heteroleptic zincates. Thus, the experiments determined not only absolute rate constants but also the branching ratios between competing reaction channels as well. In the first case, the authors investigated triarylzincates to quantify the influence of electronic effects in a Hammett‐type approach and found preferential protonation of the more electron‐rich aryl anions [180]. In the second case, they compared the tendency of ethyl and hydroxo anionic ligands toward protonation [181]. Interestingly, the latter turned out to accept a proton much more readily. High‐level coupled cluster calculations established that the energy profiles of these reactions correspond to simple double‐well potentials (Figure 8C) or slightly more complex variants thereof. Furthermore, these calculations predicted lower energy barriers for the protonation of the more electron‐rich aryl anions of the triarylzincates (relative to those for the protonation of the Ph^−^ ligand) as well as for the hydroxo ligand in the ethylhydroxo zincates (relative to those for the protonation of the Et^−^ ligand) [180, 181]. In contrast, the products resulting from the protonation of the hydroxo ligand were found to be less stable than those originating from the competing protonation of the ethyl anion. As under the given experimental conditions, the operation of a kinetic (instead of a thermodynamic) control over the competing reactions could be safely assumed, the predicted energy profiles were in qualitative agreement with the experimental findings. Note that here, in contrast to the unimolecular dissociation of the organometallic complexes discussed above, already the PESs alone permit, at least at the qualitative level, the correct prediction of the reactivity because in each case, the competing protonation reactions involve similarly tight TSs.

In the third study, Rahrt et al. probed the consecutive protolysis of triphenyl‐ and trimesitylferrate anions by 2,2,2‐trifluoro‐ and 2,2‐difluoroethanol [182]. Again, these reactions resulted in the replacement of the protonated aryl anion by the alkoxides. The special value of this study lies in its design as a blind challenge, in which the different participating groups calculated the relevant stationary points of the PESs of these reactions without knowing the experimental results. In this way, any possible bias in the theoretical calculations was excluded.

In all three studies, Rahrt et al. carried out statistical‐rate theory/master‐equation calculations on the basis of the quantum chemical computations by means of the MESMER software (see Section 3.2) [180, 181, 182]. In doing so, they simplified the reaction mechanism in that they considered only the truncated PESs up to the TSs associated with the actual proton transfer and neglected the resulting ion‐molecule complexes between the alkoxometalate anions and the newly formed hydrocarbon (or water in the case of the hydroxo protonation of the hydroxoethyl zincates) as well as the dissociation of the latter. The authors justified this approach by pointing out the rate‐limiting nature of the proton transfer included in their analysis, but did not perform any controls to test for the validity of this assumption. Most likely, the assumption is acceptable for strongly exothermic reactions, such as the protonation of the carbanionic ligands of the organozincates. However, it might be more problematic for less exothermic reactions, such as the protonation of the hydroxo ligand of the hydroxoethyl zincates. The actual proton transfer was modeled with the standard RAC‐RRKM approach. The microcanonical rate constants for the formation of the encounter complex between the reactants were calculated with the inverse Laplace transform method based on canonical collision rate constants predicted by capture theory according to Su and Chesnavich (see Section 2). In all cases, the respective reaction degeneracy was considered.

For the master‐equation calculations, Rahrt et al. assumed a temperature of *T* = 310 ± 20 K and a pressure of *p* = 0.6 × 10^−3^ mbar of the helium bath gas [180, 181, 182]. They then fitted the obtained time profiles of the population of the different species with the program Gepasi [189] to extract rate constants for the overall reactions, which they compared with the measured ones. The predicted rate constants for the protonation of the organozincate complexes matched their experimental counterparts within one order of magnitude and also showed the correct relative order [180, 181]. In most cases, the theoretical rate constants underestimated the measured ones, which argued against large errors resulting from the truncation of the considered PESs. This truncation neglects a possible additional kinetic constraint and, thus, if problematic, should lead to the prediction of too high rate constants. The absence of larger discrepancies suggests that the results of the quantum chemical computations as input for the statistical‐rate theory calculations were not grossly wrong. Such a failure would not have been expected for organozinc species with their closed d shells anyhow. In contrast, state‐of‐the‐art quantum chemical methods apparently are not yet capable of correctly predicting the barrier heights for the protonation of open‐shell organoiron complexes in all cases. As the blind challenge showed, the five different employed methods calculated reaction barriers, which spread over quite a large energy range (up to > 70 kJ mol^−1^ in the extreme) [182]. Accordingly, the corresponding theoretical rate constants also varied considerably and deviated from the experimental ones by up to several orders of magnitude. None but one of the different approaches succeeded in predicting the correct order of the rate constants of the probed reactions.

The presented examples show the potential of statistical‐rate theory calculations for the ab initio prediction of rate constants for bimolecular reactions involving organometallic ions. For systems exhibiting a limited mechanistic complexity and being amenable to accurate quantum chemical calculations, the kinetic calculations can reproduce measured rate constants within one order of magnitude. In many cases, such an accuracy is already sufficient for confirming or refuting mechanistic hypotheses or for assessing the reliability of quantum chemical calculations. With respect to the latter, the discussed work has clearly demonstrated the difficulties of the accurate treatment of open‐shell species. For the modeling of reactions involving only tight TSs whose energy lies significantly below the entrance channel, the details of the underlying PESs are less important. Instead, here the barrierless capture process needs to be described as accurately as possible. To this end, the PST/PSL‐RRKM, VTST, or SACM/SSACM approaches can be applied. Furthermore, the effect of deactivating collisions between energized ionic intermediates and the bath gas present in quadrupole ion traps lacks a thorough understanding. Currently, empirical models are used to account for this effect, thereby compromising the a priori nature of the whole approach. To improve our understanding in this regard and for a more rigorous testing of the theoretical methods in general, experiments over a larger temperature range would be very welcome. Such experiments have been performed extensively with selected‐ion flow tube instruments [190, 191, 192, 193, 194, 195, 196, 197]. They should also be feasible with quadrupole‐ion traps, provided the latter were substantially modified to allow for proper temperature control and adjustment [198, 199].

Finally, we would like to emphasize that the application of statistical rate theory to the prediction of bimolecular rate constants of ion‐molecule reactions goes beyond the examples from organometallic chemistry covered here. Several studies on metal‐free systems have been reported, which also included a correction for quantum‐mechanical tunneling effects, thereby further advancing and refining the theoretical methodology [186, 187, 188].

### 4.5 Evaluation of Statistical‐Rate Theory Approaches for Gas‐Phase Ion Reactions

Sophisticated experiments on the dissociation of the *n*‐butylbenzene radical cation and benzylpyridinium ions have provided valuable benchmark data for assessing the performance of different statistical‐rate theory approaches for gas‐phase ion reactions (see Sections 4.1 and 4.2). These approaches have been used for fitting the experimental data to extract reaction threshold energies *E*_0_. A validation of the different theoretical methods then becomes possible by comparing the thus obtained *E*_0_ values with independently determined reference energies. From the analysis of these systems, the following lessons have been learned:

(i) Heterolytic bond‐cleavage reactions: similar to dissociations of noncovalently bound ion‐molecule complexes (cf. Section 2), these elementary reactions proceeding via typical loose TSs are well described by PST/PSL‐RRKM calculations.
(ii) Homolytic bond‐cleavage reactions: the TSs of these elementary reactions also have a loose character, but not as pronounced as those of heterolytic bond‐cleavage reactions owing to a more anisotropic interaction potential between their fragments. Due to this intermediate and more complex situation, neither RAC‐RRKM nor PST/PSL‐RRKM calculations will achieve an adequate description. Both the VTST and the SACM approaches can cope with such cases but are much more demanding than RAC‐ or PST/PSL‐RRKM calculations and, thus, not suitable for routine applications. A possible solution to this problem is the SSCAM method, which combines PST/PSL‐RRKM calculations with rigidity factors from simplified expressions. The latter can be derived from SACM computations (optimized with the aid of experimental results) for the same or related systems.
(iii) Fragmentation reactions featuring isomerization/rearrangement before the dissociation: these reactions involving typical tight TSs can be satisfactorily modeled by RAC‐RRKM calculations in a straightforward manner. However, given the multistep nature of these reactions, merely considering the tight‐TS bond rearrangement for calculating their rate constants is only appropriate if it is clearly the rate‐determining step. In the case that the rearrangement barrier is well below the energy of the fragmentation products, the dissociation step should not be neglected within the kinetic modeling. At the same time, for such a situation, the entropically less favorable rearrangement step should not be disregarded either, just because it is not the energetic bottleneck.

These general considerations should hold not only for the dissociation of organic ions but also for that of their organometallic counterparts. For the latter, the correct characterization by quantum chemical methods is more demanding than for simple organic systems, for which reason the prediction of the rate constants for their fragmentation is likely to be less accurate. However, the very limited number of available experimental benchmark data on organometallic ions does not permit a rigorous test of this conjecture. Irrespective of the system investigated, the validity of the theoretical modeling of the experimentally observed dissociation reactions would benefit from a more precise determination of the experimental time scale *τ* for different instruments. In many cases, not even the uncertainties of the assumed time scales have been quantified. Likewise, more detailed information on the energy distributions of the probed ions would be helpful.

In comparison to unimolecular reactions of organometallic ions, their bimolecular analogs are more complex and always consist of several elementary steps, including the actual collision events yielding the ion‐molecule encounter complexes. For estimating the microcanonical rate constants of this association, simple models are available and frequently relied upon, although VTST or SACM/SSACM calculations would probably furnish a better quantification of the ion‐molecule capture. In quadrupole‐ion traps, the reactants of ion‐molecule reactions typically have thermal and, thus, well‐defined energy distributions. In contrast, the energy distributions as well as the population of rotational states of the ionic intermediates transiently formed in these reactions are not known precisely. This situation is complicated by the effect of collisions between the ions and the neutral bath gas. The transfer of energy during these collisions is not well understood and treated by purely empirical models in statistical‐rate theory/master‐equation calculations. This poor understanding is problematic for reactions involving intermediates with relatively long lifetimes, which will then experience numerous collisions. Irrespective of this complication in their theoretical description, mass‐spectrometric experiments on ion‐molecule reactions allow for the determination of rate constants with relatively good precision. The measured rate constants can then be compared with statistical‐rate theory predictions in a quantitative manner. These comparisons show that for the reactions of medium‐sized or large open‐shell organometallics, a satisfactory agreement between experiment and theory may not necessarily be reached due to difficulties in the calculation of accurate barrier heights in these cases. For the reactions of closed‐shell organometallics, experimental and theoretical rate constants typically agree within one order of magnitude or better, which appears much more encouraging and, thus, suggests that statistical‐rate theory calculations can indeed be applied for testing the validity of quantum chemical calculations and discriminating between different mechanistic pathways of these systems. However, most of the probed reactions are exothermic with submerged barriers. Reactions with significant energy barriers (≥ 10 kJ mol^−1^ above the entrance channel) would proceed too slowly for their convenient monitoring in the experiments. The faster the reactions are, the more closely they will approach the limit of the collision rate, which exerts a leveling effect and thereby diminishes the sensitivity of the calculated rate constant on the predicted reaction barrier. This leveling effect possibly explains why O’Hair and coworkers could theoretically reproduce the rate constants of the faster reactions, which they analyzed [177, 178, 179], more accurately than Rahrt et al. were able to do for the slower reactions, on which they focused by probing systems with less negative reaction barriers (relative to the reactants) [180, 181, 182]. Clearly, for a more rigorous testing of the computational methods used for the bimolecular rate constant predictions, i.e., quantum chemical calculations and statistical‐rate theory approaches, slower ion‐molecule reactions should be probed. For this purpose, it would also be quite helpful to study ion‐molecule reactions at different temperatures. The determination of rate constants as a function of temperature is standard practice in the kinetic analysis of neutral reactions, but it is not applied routinely for ion‐molecule reactions, apart from the extensive work of Viggiano, Shuman, Ard, and colleagues [57].

## 5 Summary and Outlook

Statistical rate theory forms a well‐established framework for the quantitative description of uni‐ and bimolecular gas‐phase ion reactions. Its earlier applications focused mainly on the fitting of experimental data for the extraction of the quantities of interest, such as reaction threshold energies. Those earlier studies allowed for the rigorous testing of different approaches. As a result of these efforts, by now a fairly good understanding has been reached of which methods should be applied to which cases. At the same time, numerous programs are available for performing statistical‐rate theory calculations in a convenient manner without expert knowledge in scientific computing. Together with the wide availability of software for quantum chemical calculations, the users have all the means for the ab initio prediction of rate constants at their command. Modern quantum chemical methods are sufficiently accurate for correctly characterizing the reactions of small and medium‐sized closed‐shell organic and organometallic ions. Accordingly, the rate constants computed for these systems on the basis of the results of such quantum chemical calculations can be expected to achieve qualitative and also at least semiquantitative agreement with the experimental rate constants. The direct comparison of experimentally measured branching ratios and/or absolute rate constants with theoretical predictions for different reaction pathways then allows for the discrimination between the latter and, thus, affords mechanistic insight of critical importance. Importantly, the experimental observables required for this approach can be obtained with simple commercial or only slightly modified instruments. The feasibility of this strategy has already been demonstrated for a number of examples.

For further enhancing the attainable accuracy of the theoretically predicted rate constants, several improvements would be instrumental. On the side of the experiment, the time scale of CID experiments should be determined more precisely to allow for a more accurate calculation of kinetic shifts and experimentally relevant reaction rate ranges. For the analysis of ion‐molecule reactions, experiments at different temperatures would provide valuable information on the temperature dependence of the rate constants. On the side of the quantum chemical calculations, improved and/or more efficient methods for the reliable description of open‐shell (organometallic) ions are needed, not only in the present context. With respect to statistical‐rate theory/master‐equation computations, a better understanding of collisional energy transfer under multiple‐collision conditions, such as in quadrupole ion traps, would be advantageous. Moreover, the available software for these calculations would benefit from a more widespread implementation of the more refined methods developed for modeling barrierless elementary steps (PST/PSL‐RRKM, VTST, SSACM). Finally, the recent advances in molecular dynamics simulations render this approach a possible alternative or complementary tool for the prediction and theoretical analysis of gas‐phase ion reactions.

## 6 Author Contributions

**Thomas Auth**: conceptualization, writing – original draft, writing – review & editing. **Konrad Koszinowski**: conceptualization, writing – original draft, writing – review & editing, funding acquisition.

## Funding

This study was supported by Deutsche Forschungsgemeinschaft (KO 2875/15‐1).

## Conflicts of Interest

The authors declare no conflicts of interest.

## Data Availability

The manuscript does not contain any original data.
